# Identification and targeting oxidative phosphorylation/glycolysis to overcome anti-CSF-1R therapy resistance in glioblastoma

**DOI:** 10.1038/s41419-025-08288-3

**Published:** 2025-12-10

**Authors:** Cheng Miao, Zehua Ding, Jiaxing Wu, Qi An, Ya Shu, Haifeng Jiang, Panpan Gao, Ruoqiao Chen, Xiao Qian Chen

**Affiliations:** 1 Department of Obstetrics and Gynecology, Shanghai Key Laboratory of Maternal Fetal Medicine, Shanghai Institute of Maternal Fetal Medicine and Gynecologic Oncology, Shanghai First Maternity and Infant Hospital, School of Medicine, Tongi University, Shanghai, China; 2 Department of Pathophysiology, School of Basic Medicine, Tongji Medical College, Key Laboratory of Neurological Diseases, Ministry of Education; Hubei Provincial Key Laboratory of Neurological Diseases, Huazhong University of Science and Technology, Wuhan, China; 3 Department of Pharmacy, The First Affiliated Hospital of Yangtze University, Jingzhou, China; 4 Department of Pharmacology and Toxicology, Michigan State University, East Lansing, MI USA

**Keywords:** Cancer therapeutic resistance, Cancer microenvironment, Cancer metabolism

## Abstract

The standard care of glioblastomas (GBM) confers limited survival benefit for patients due to the rapid tumor recurrence. Targeting tumor-associated macrophages/microglia via colony-stimulating factor 1 receptor (CSF-1R) inhibition is potentially effective in suppressing GBM recurrence. However, clinical trials of CSF-1R inhibitors failed to achieve their goal due to GBM resistance to anti-CSF-1R therapy. Here, we identified and verified key resistance mechanisms of anti-CSF-1R therapy by translatome profiling-combined analyses. To solve above problem, we have established a highly stable and refractory mouse G422^TN^-GBM model, in which temozolomide (TMZ) is the most effective monotherapy but can only slightly extend animal survival. To identify effective resistance mechanism of anti-CSF1R therapy in GBM, we first apply the Translating ribosome affinity purification (TRAP) RNA-sequencing techniques in GBM tissues, which have previously used in neuroscience. TRAP-seq identified oxidative phosphorylation/glycolysis as anti-CSF1R therapy resistance mechanism, and it’s combined with Cancer Therapeutics Response Portal (CTRP) identified piperlongumine (PL) or vorinostat (SAHA) as targeting drugs. PL or SAHA enhanced PLX3397 efficacy by reversing oxidative phosphorylation/glycolysis dysregulation in vitro and in vivo. The triple combination of PLX3397, TMZ, and PL/SAHA significantly improved survival in G422^TN^-GBM mice. In conclusion, targeting oxidative phosphorylation/glycolysis by PL or SAHA prominently improves therapeutic efficacy of PLX3397 + TMZ in GBM, which deserves priority for clinical trials. Our study also reveals that translatome profiling is efficient for uncovering drug-resistant targets.

## Introduction

Glioblastoma multiforme (GBM) is the most malignant primary brain cancer, with an average survival time of around 12 months [1–3]. The standard treatment involves surgical resection followed by radiotherapy and concurrent temozolomide (RT/TMZ), which modestly extends median survival by 2 to 3 months and achieves 5-year survival in merely 9.8% patients [3, 4]. The high level of invasiveness and heterogeneity of GBM limits the effectiveness of current therapies, and tumors inevitably recur due to multidrug resistance [5, 6].

Tumor-associated macrophages & microglia (TAMs) are major immune-suppressive cells in tumor microenvironment that promote GBM progression [7, 8]. Colony Stimulating Factor 1 Receptor (CSF-1R), a tyrosine kinase receptor, is widely expressed in monocytes, macrophages and microglia and is considered a key regulator for GBM recurrence [9, 10]. Targeting TAMs through CSF-1R inhibition has emerged as a promising therapeutic strategy to prevent GBM recurrence [11, 12]. PLX3397 (i.e., pexidartinib), an FDA-approved CSF1R inhibitor, has shown therapeutic effects for tenosynovial giant cell tumor [13]. However, in GBM Phase II trials, PLX3397 and other anti-CSF-1R therapies all failed to extend patient survival either as a monotherapy or combined with radiotherapy and TMZ in newly diagnosed GBM [14–16], although anti-CSF-1R monotherapy effectively extends animal survival in PDGF-B driven murine glioma (PDG) model [11, 17] or human GBM cell-derived xenografts model [18]. Therefore, finding the underlying resistance mechanism in anti-CSF-1R therapy in GBM is crucial for further clinical trials [15]. Previous studies have suggested that CSF-1R mutation (e.g., G795A) in macrophages or IGF-1R/PI3K in PDG glioma cells contribute to resistance to CSF-1R inhibitors [19, 20]. To further explore the resistance mechanism of anti-CSF-1R therapy in GBM, we applied translatome profiling in a highly refractory G422^TN^-GBM mouse model, which accurately replicates the therapeutic responses and tumor recurrence of human GBM [21–26].

It is well known that the mRNA levels measured by bulk RNA-seq or scRNA-seq in cancer cells do not accurately reflect their translation level due to mRNA instability/regulation [27–30]. Translating ribosome affinity purification (TRAP) technology catches the mRNA in ribosome in situ, which accurately represents its translation level in specific cellular contexts. By performing TRAP-seq in L10a-eGFP-overexpressing G422^TN^-GBM cells, we can measure the level of mRNAs translated in the ribosome in GBM cells in vivo. This approach overcomes the limitations of bulk RNA-seq or scRNA-seq techniques, enabling a more accurate exploration of cancer progression mechanisms, drug resistance, and the evaluation of new anticancer drugs [30].

In this study, our snRNA-seq data revealed that CSF1/IL34-CSF1R interaction was the strongest between G422^TN^-GBM cells and microglia, which indicated a worse prognosis. Blocking CSF-1R signaling by PLX3397 effectively killed TAMs, suppressed GBM development initially, but tumor relapsed rapidly. Combining TRAP-seq data in G422^TN^-cells in the brain and data from the Cancer Therapeutics Response Portal (CTRP) database, we identified piperlongumine (PL) and vorinostat as the top 2 drugs to overcome PLX3397-therapy resistance. Indeed, PL or vorinostat significantly improved efficacy of PLX3397 therapy by reversing the elevated glycolysis and the reduced oxidative phosphorylation. Furthermore, PL or vorinostat combined with PLX3397-based TMZ chemotherapy prominently extended animal survival in the incurable G422^TN^-GBM model, suggesting effective regimens for GBM clinical trials.

## Methods

### Animals

Adult male C57 mice (22 ± 2 g) and Kunming mice (20 ± 2 g) were purchased from Hubei Biont Biological Technology Co., Ltd. These animals were maintained at the Animal Center of Huazhong University of Science and Technology under a 12-h light-dark cycle, with unrestricted access to water and food.

### Orthotopic glioma model

C57 mice were anesthetized by intraperitoneal injection of chloral hydrate (350 mg/kg) and xylazine (10 mg/kg). Using a 10 μl Hamilton syringe, 2 × 10^4^ G422^TN^ tumor cells (in 1 μl PBS) were injected into the right striatum of the mouse brain (coordinates: 0.5 mm anterior and 2.0 mm lateral from the bregma, and 3.5 mm deep from the skull surface) based on the brain atlas.

### Frozen section preparation and immunofluorescence staining

Immunofluorescent staining was performed as previously described [31, 32]. Mice were perfused with cold 0.9% saline, followed by perfusion with cold 4% paraformaldehyde. Subsequently, the brain tissue was fixed in 4% paraformaldehyde at 4 °C for 6 h. After fixation, the brain tissue was sequentially placed in 20 and 30% sucrose solutions until sinking. The brain tissue was embedded in OCT compound, and frozen sections (20 μm) were prepared and then incubated with primary and secondary antibodies (1:200, Alexa Fluor 594, Jackson ImmunoResearch). The primary antibody was anti-IBA-1(1:500, ab178846, Abcam). After mounting, the slides were stored in a 4 °C refrigerator and promptly imaged and photographed under the fully automated slide scanner SV120 (Olympus).

### Drug treatment

#### In vivo

PLX3397 (MedChemExpress MCE, China) was dissolved in 10% Tween-80, 10% DMSO and 80% saline water and administered to mice (50 mg/kg/d) daily via intraperitoneal injection [17, 33, 34]. Piperlongumine (Selleck Chemicals, China) was dissolved in Tween-80/water (10/90, v/v) at a final concentration of 0.5 mg/ml, and administered to mice (5 mg/kg/d) daily via intraperitoneal injection. Vorinostat (MedChemExpress MCE, China) was dissolved in 5% DMSO, 40% PEG 300, 5% Tween-80 and 50% saline water and administered to mice (50 mg/kg/d) daily via intraperitoneal injection [35, 36]. Temozolomide (TMZ, Abmole, China) was prepared as a 5 mg/ml solution in 0.5% sodium carboxymethyl cellulose (CMC-Na) and administered to mice at a dosage of 30 mg/kg daily through oral gavage [37].

#### In vitro

PLX3397 (MedChemExpress MCE, China) was dissolved in DMSO solution as a storage solution at a final concentration of 1 mM. Piperlongumine (Selleck Chemicals, China) was dissolved in DMSO solution as a storage solution at a final concentration of 1 mM. Vorinostat (MedChemExpress MCE, China) was dissolved in DMSO solution as a storage solution at a final concentration of 1 mM. Temozolomide (TMZ, Abmole, China) was dissolved in DMSO solution as a storage solution at a final concentration of 5 mM. The stock solution was diluted with cell culture medium into a working solution prior to use.

### GFP fluorescence imaging

For mice in the control and drug treatment groups, perfusion and tissue collection were performed. The mouse brain tissue was placed on black cardboard and positioned in the small animal in vivo imaging system (Lago X), with the excitation wavelength, emission wavelength, and exposure time set. After imaging, the images were saved, and image analysis was performed using Amiview software (Spectral Instruments Imaging Company, USA) to statistically analyze the light density values in the regions of interest (ROI).

### Hematoxylin-eosin (H&E) staining and immunohistochemistry (IHC) staining

Brain slices embedded in paraffin were utilized for H&E staining and IHC analysis, following the established protocols [38]. In summary, 4 μm-thick sections of brain tissue were processed by removing paraffin, rehydrating, inhibiting endogenous peroxidases, retrieving antigens, and blocking with 5% BSA. They were then incubated with primary and secondary antibodies, and developed with diaminobenzidine tetrachloride to produce a colored reaction (Polink-1 HRP DAB Detection System from ZSGB-BIO, China). Primary antibodies were anti-Ki67 (1:200, ab16667, Abcam, UK), anti-CD3 (1:2000, ab237721, Abcam, UK), anti-cleaved caspase 3(1:500, ab32042, Abcam, UK), anti-Vimentin(1:200, ab92547, Abcam, UK), anti-CD31(1:500, ab9498, Abcam, UK), anti-P-PI3K(1:200, #17366,Cell Signaling Technology), anti-P-AKT(1:100, #4060, Cell Signaling Technology), anti-cytochrome C(1:200, 10993-1-AP, Proteintech, China), anti-GLUT1(1:200, 21829-1-AP, Proteintech, China), anti-HK2(1:200, 66974-1-Ig, Proteintech, China), anti-LDHA(1:200, 21799-1-AP, Proteintech, China), anti-MCT4(1:200, 22787-1-AP, Proteintech, China), anti-MT-ATP6(1:200, A17960, ABclonal, China), anti-PKM2(1:200, 15822-1-AP, Proteintech, China), anti-CD206(1:200, 18704-1-AP, Proteintech, China), anti-CD68(1:200, 28058-1-AP, Proteintech, China), anti-MT-ND1(1:200, Servicebio, GB113284, China), anti-MT-CO1(1:100, ab14705, Abcam, UK) and anti-SDHA(1:100, ab14715, Abcam, UK). After mounting, imaging was performed using the fully automated slide scanner SV120 (Olympus). Statistical analysis was based on the average of at least 3 brain tissue sections from each mouse, with at least 3 mice per group.

### Terminal deoxynucleotidyl transferase-mediated dUTP nick end labeling (TUNEL) staining

In order to elucidate the impact of PLX3397 on apoptosis of tumor cells, the TUNEL cell apoptosis detection kit (Beyotime Biotechnology, C1088) was used to stain frozen sections of mouse brain tissue. TUNEL staining positive signals appeared as green fluorescence, representing apoptotic cells.

### Bulk-RNA sequencing (RNA-seq)

RNA-seq was employed to profile genome-wide transcriptional landscapes across tumor. Total RNA was isolated from homogenized samples, followed by quality assessment using RNA integrity number. Strand-specific cDNA libraries were then constructed through fragmentation, reverse transcription, and adapter ligation, followed by sequencing on Illumina NovaSeq platforms. Downstream analyses typically encompassed differential expression testing and functional enrichment (GO, KEGG).

### Cell culture of G422^TN^ and BV2 cell lines

All cells were cultured in a CO_2_ incubator at 37 °C. The G422^TN^ tumor cells were highly invasive cells purified from the G422 tumors in situ in mice and passed only in vivo. Primary G422^TN^-cells were cultured in RPMI 1640 (Servicebio, China) supplemented with 10% FBS (Gibco) and 1% Penicillin–Streptomycin Solution. The BV2 cell lines were purchased from Sunn Biotechnology Co., Ltd. (SNL-155, China), and cultured in DMEM (Gibco, USA) supplemented with 10% FBS (Gibco, USA) and 1% Penicillin–Streptomycin Solution. The cultures were used within 30 cell passages.

### Cell Counting Kit 8 (CCK8) assay

This assay is based on the conversion of WST-8 (Beyotime Biotechnology, C0037) by cellular dehydrogenases into a water-soluble formazan dye, which changes color and is quantified by absorbance measurement. G422^TN^ tumor cells were treated with PLX3397, PL, or vorinostat for 24 or 48 h. The CCK8 assay then determined cell viability and cytotoxicity and explored suitable concentrations of drugs in vitro.

### Propidium iodide staining

Cells were first fixed with 4% paraformaldehyde treatment, then permeabilized with 0.1% Triton X-100 to allow PI (Beyotime Biotechnology, C1352S) entry, before incubated with PI solution (50–100 μg/mL), which stains DNA for 30 min. The PI fluorescence is analyzed using a flow cytometer and microscope.

### Lentiviral transfection

G422^TN^-cells were infected with L10aGFP-expressing lentivirus using the transfection method previously described [22]. The lentivirus (VSVG-Lentiviral-UbiC-GFPL10a-WPRE) was purchased from Shanghai Taitool Bioscience Co., Ltd (Shanghai, China). The appropriate amount of lentivirus (Multiplicity of Infection (MOI): 10) was added according to the instructions. The successfully infected G422^TN^ tumor cells were injected subcutaneously into Kunming mice, and tumor formation was closely monitored. After stable passage in subcutaneous tissue, the cells, named L10aGFP-G422^TN^, were used for subsequent animal experiments.

### Western-blot

Tumor lysates from each group were separated by gel electrophoresis, transferred to a membrane, and then incubated with GFP antibody (1:1000, 50430-2-AP, Proteintech Group). After washing, a secondary antibody (1:2000, GB23303, Servicebio) was applied, and the protein was visualized using a detection system. The image was saved and analyzed with the ImageJ software.

### Translating ribosome affinity purification (TRAP) and RNA sequencing

Affinity purification of translating ribosomes was minor modified from a standard protocol [39]. GFPL10a-G422^TN^ cells were injected into mouse striatum. A week later, tumor-containing brain areas were dissected at 4 °C, pooled from three mice per sample (three replicates per group), and washed in cold dissection buffer for blood removal. Samples were homogenized in a pre-chilled buffer with 20 mM HEPES, pH 7.4, 10 mM MgCl2, 150 mM KCl, 0.5 mM DTT, 100 mg/ml cycloheximide, 200 units/ml RNasin and Superasin, using a Dounce homogenizer. Homogenates were centrifuged at 2000 × *g* for 10 min at 4 °C to remove pellets. 1%NP-40 and 0.5 mM 1,2-diheptanoyl-sn-glycero-3-phosphocholine were added to the supernatant, followed by centrifugation at 20,000 × *g* min to pellet insolubles. 5% supernatant aliquot was saved as the “Input” fraction, and GFP antibodies were added to the remainder for 4 h at 4 °C. Protein A + G magnetic beads were added post-incubation, and the mixture was incubated for 16–18 h at 4 °C. Beads were then washed with high-salt buffer, and the supernatant was collected as the “IP” fraction after magnet separation. RNA was extracted from both “Input” and “IP” fractions using an RNA isolation kit (Qiagen Kit 74004), and samples were sent to Shanghai Bohao Biotechnology for quality inspection and sequencing analysis.

### Transmission electron microscopy

In order to visually observe the morphology of tumor cell mitochondria and assess the mitochondrial function of tumor cells, C57 mice were orthotopically injected with glioma cells and subjected to drug treatment. Tumor tissues were cut into approximately 1 mm^3^ pieces, which were rapidly placed in cold 2.5% glutaraldehyde fixative (G5882, Sigma-Aldrich). Subsequently, the samples were fixed in 1% osmium tetroxide (Sigma-Aldrich, O5500) for 1.5 h and dehydrated in a graded ethanol series. The samples were then infiltrated and embedded in Epon Resin and polymerized at 60 °C for 12 h. The tissue was sectioned into 60 nm ultrathin slices and stained with uranyl acetate and lead citrate. Following staining, observation and photography of mitochondrial morphology were conducted under a transmission electron microscope (Hitachi HT7700), and subsequent analysis was performed.

### Collection of conditional culture medium

BV2 cells were stimulated with DMSO or PLX3397 for 24 h. The culture medium was discarded and replaced with DMEM containing 0.5% BSA, followed by another 24-h incubation. The cell culture medium collected after treatment with DMSO or PLX3397 is known as BV2-conditioned medium (BV2-CM or Plx-BV2-CM).

### Measurement of cellular ATP level

In order to determine the impact of drug treatment on the oxidative energy metabolism of glioma cells, the ATP Assay Kit (Beyotime Biotechnology, S0026) was utilized to assess the effect of drug treatment on ATP generation in glioma cells. Following a 24-h drug treatment of the cells, ATP detection lysis buffer was added to release ATP, and luminescence values (RLU) were measured using a chemiluminescence analyzer (luminometer).

### Measurement of cellular lactate level

Lactate levels in various groups of tumor cells were determined using a lactic acid assay kit (A019-2-1, Nanjing Jiancheng Bioengineering Institute). G422^TN^ tumor cells were collected from each group, on which the ultrasonic lysis was performed. Following the instructions of the assay kit, lactate was extracted from the tumor cells step by step. Luminescence values were measured using a chemiluminescence analyzer (luminometer).

### Reactive oxygen species (ROS) generation

ROS was measured after 24 h of drug treatment. To measure intracellular reactive oxygen species (ROS) production, G422^TN^ cells were loaded with the fluorescent marker, 2,7-dichlorodihydrofluorescein diacetate (H2DCF-DA, Elabscience Biotechnology Co. Ltd, China), in DMEM/MEM without phenol red for 30 min and then washed with PBS. After the incubation was completed, the cells were washed with PBS and immediately observed under a fluorescence microscope. For each sample, the mean fluorescence intensity of the same number of cells was determined to represent the intracellular ROS production.

### Mitochondrial membrane potential (MMP) analysis

To determine the effect of drug treatment on oxidative energy metabolism in glioma cells, the mitochondrial membrane potential assay kit (Beyotime Biotechnology, C2006) with JC-1 was used. After the detection was completed, the expression of red and green fluorescence was observed under a fluorescence microscope, and the proportion of mitochondria depolarization was measured by the relative ratio of red and green fluorescence.

### Data collection and preprocessing

Glioma patients’ RNA expression data and corresponding clinical information were downloaded from the American Cancer Gene Atlas Project (TCGA) and the Chinese Brain Glioma Gene Atlas Project (CGGA). The glioma datasets GSE131660 [40] and GSE110948 were downloaded from Gene Expression Omnibus. The SnRNA-seq dataset PRJNA1043813 was downloaded from the Sequence Read Archive. For single-cell RNA sequencing data, GSE242790 [41] and SnRNA sequencing data, cell clusters were identified using the FindClusters, infercnv and copykat tools. Marker genes were used to identify all the different cell clusters for annotation. The UMAP function was used for visualization.

### Gene set and differential metabolite enrichment analysis

All pathway enrichment analyses were based on the Kyoto Encyclopedia of Genes and Genomes (KEGG) and Gene Ontology (GO) database resources. Besides, gene set enrichment analysis (GSEA) software (version 4.3.2) was used to analyze the potential signaling pathways. Gene sets of glycolysis and oxidative phosphorylation signature were downloaded from Molecular Signatures Databaes. The gene sets’ scores were calculated based on the transcriptome profiling data via the single-sample Gene Set Enrichment Analysis (ssGSEA) algorithm (R package “GSVA”)

### Drug sensitivity analysis

IC50 values of 481 small molecules across 1001 cell lines and corresponding mRNA gene expression data were collected from CTRP. Pearson correlation analysis was used to reveal the relationship between gene mRNA expression and drug IC_50_ values. To adjust for multiple comparisons, the false discovery Rate method was used to correct *p*-values.

### Survival analysis

Patients were categorized into a “low survival group”, if their survival time was below the 75th percentile of the total follow-up duration, and the remaining patients were classified as the “high survival group.”

Kaplan-Meier estimation was used for survival analysis, which was compared by log-rank (Mantel Cox) test. When the survival curves crossed, Landmark test was used. R package survminer (version 0.4.2) was used to calculate the critical value of each group.

### Statistical analysis

Two-tailed unpaired t-test was used to analyze the differences between two unpaired groups. Animal survival was analyzed by the Kaplan-Meier estimate and compared using a log-rank (Mantel Cox) test. One-way analysis of variance with post-hoc Dunnett’s test was used to compare one-factor variable experiments among multiple groups. All values were reported as mean ± SEM. Levene’s test was performed between the comparison groups to demonstrate that the variances are similar. Differences were considered significant at a value of *P* < 0.05. Descriptions for statistical analysis were shown in each corresponding Fig. legend. Statistical analysis was performed using R software (version 4.0.2) and GraphPad Prism software.

## Results

### CSF1 signaling in GBM cells and TAMs plays a major role in GBM progression

**Fig. 1:**
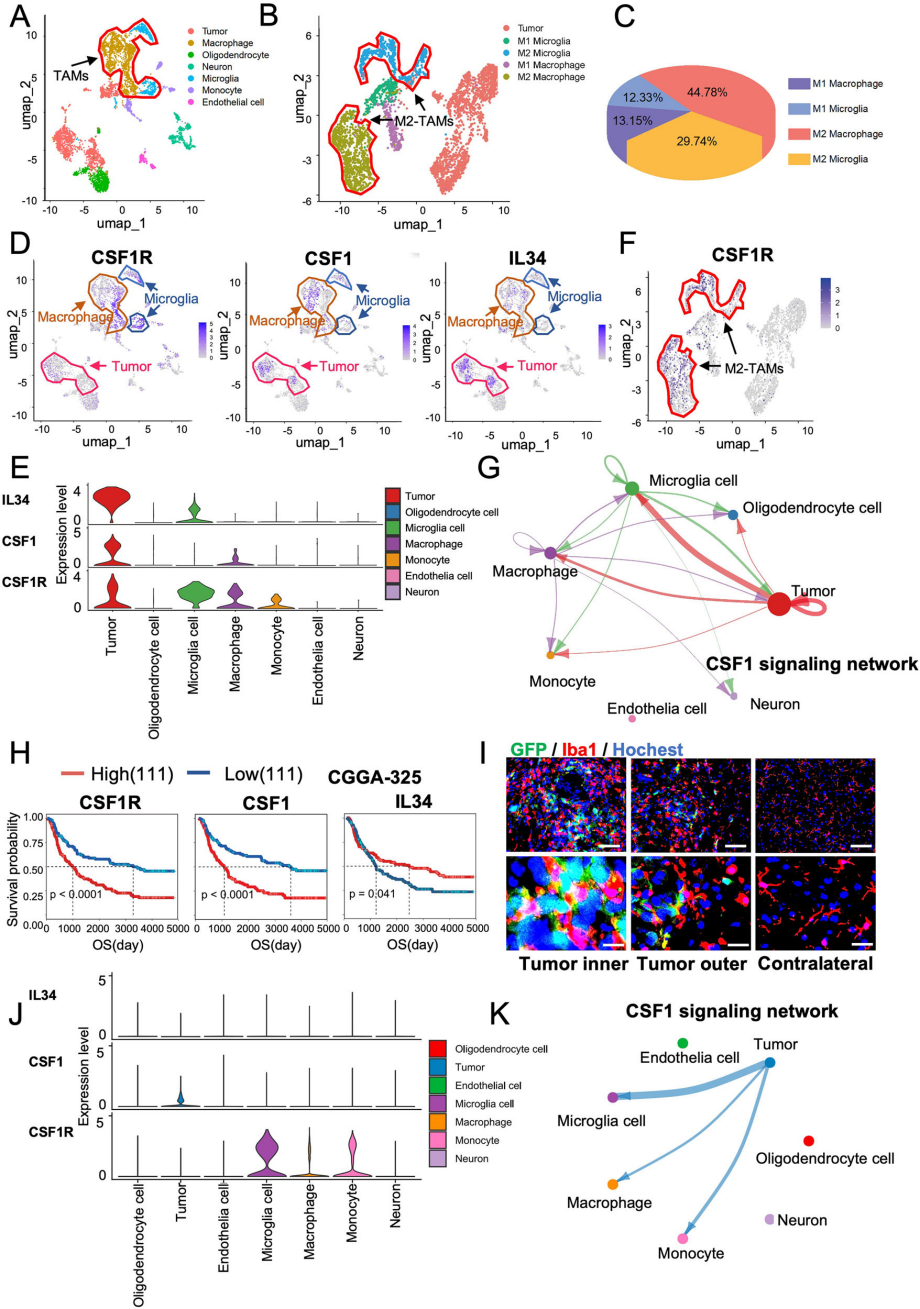
CSF1 signaling pathway plays a significant role in the progression of gliomas. **A** tSNE plot showing all cell types identified in human scRNA-seq dataset (GSE242790) (*n* = 3). **B** tSNE plot showing M1, M2 subtypes of macrophage/microglia and tumor cells in the scRNA-seq dataset (*n* = 3/group). **C** Pie charts visualizing the relative population of M1, M2 subtype of macrophage/microglia. **D** tSNE plot showing IL34, CSF1, CSF1R expression in all cell types across human scRNA-seq dataset (*n* = 3). **E** Violin plot showing expression of IL34, CSF1, CSF1R in all cell types. **F** tSNE plot showing CSF1R expression in macrophage/microglia and tumor cells across scRNA-seq databases. **G** Network diagram illustrating the interactions between tumor and environment cells of CSF1 signaling pathway. **H** Patient survival in high CSF1R expression group vs. low CSF1R expression group (left), high CSF1 expression group vs. low CSF1 expression group (middle), high IL34 expression group vs. low IL34 expression group (right) of CGGA databases. **I** Immunofluorescence staining of Iba1and Hoechst of mouse G422^TN^-GBM tumors. Scale bar, 100 μm (up), 20 μm (down). **J** Violin plot showing expression of IL34, CSF1, CSF1R in all cell types in G422^-TN^ tumor snRNA-seq dataset. **K** Network diagram illustrating the interactions between tumor and environment cells of CSF1 signaling pathway.

To determine the function of CSF-1R signaling in TAMs in GBM, we utilized human glioma scRNA-seq data. The results clearly distinguished TAMs that include both macrophages and microglia in GBM tissues (Fig. 1A). Further analyses showed that M2 subtypes of TAMs (M2-TAMs) were the predominant (74.53% of M2 over 25.47% of M1) in human GBM (Fig. 1B, C), consistent with the roles of M2-TAMs in tumor growth promotion and immune suppression [42, 43]. Then, we analyzed CSF1-CSF1R signaling among GBM cells and tumor microenvironmental cells. scRNA-seq showed that the CSF-1R ligands (CSF-1 and IL34) [12] as well as CSF-1R were primarily expressed in tumor cells and TAMs, with the highest expression of IL34/CSF-1 in GBM cells and the highest expression of CSF-1R in microglia (Fig 1D, E). Specifically, CSF-1R expression in M2-TAMs was much higher than TAMs-M1 (Fig. 1F). Cell communication analysis of the CSF1 signaling network revealed that most CSF-1/IL-34 signals were emitted by tumor cells, which were primarily received by TAMs, while the tumor cells also received autocrine CSF-1/IL-34 signals (Fig. 1G and Supplementary Fig. 1B, C). Such evidence suggests a major role of microglia in CSF1R-mediated GBM progression or recurrence. Finally, we analyzed the relationship between CSF1R/CSF1/IL34 expression and patient overall survival (OS), and the results showed that higher CSF1R/CSF1, as well as lower IL34 levels were significantly correlated to shorter OS in GBM patients (Fig. 1H and Supplementary Fig. 1A), supporting anti-CSF1R therapy in GBM.

To obtain reliable preclinical data for evaluating drug efficacy and mechanisms, we have previously successfully established a highly refractory murine G422^TN^-GBM model resistant to standard surgery/radiotherapy/TMZ therapies with 100% rapid tumor recurrence [23]. On day 7 of tumor growth, a large amount of hyper-activated TAMs (Iba1^+^, red) existed in both G422^TN^-tumor internal and marginal regions (Fig. 1I). Consistent with human GBM data, snRNA-seq of G422^TN^-tumor also revealed a major CSF1-CSF1R crosstalk between cancer cells and microglia (mainly M2 subtype) (Fig. 1J, K). Taken together, our G422^TN^-GBM model mimics well the CSF1-CSF1R signaling and therapeutic responses in human GBM.

### PLX3397 monotherapy initially prominently inhibits G422^TN^-tumor growth but only slightly extends survival

**Fig. 2:**
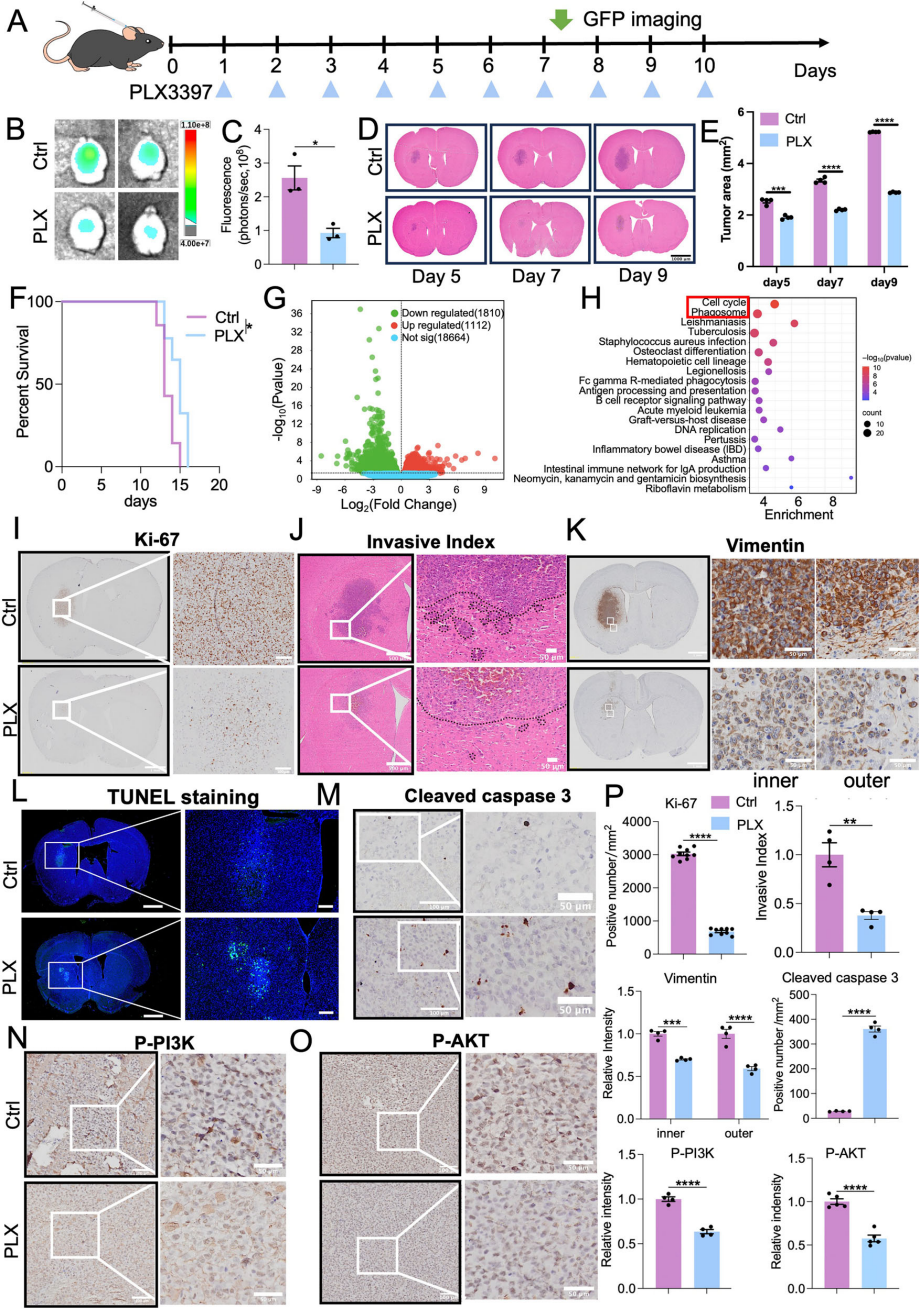
CSF-1R inhibitor PLX3397 prominently inhibits G422^TN^-GBM growth via targeting cell cycle. **A** Schematic diagram illustrating PLX3397 (Plx) monotherapy starting on day 1 post G422^TN^-cell inoculation (p.i.) (100 mg/kg/d, p.o., q.d., 10 doses). **B** **C** Representative GFP fluorescence images and statistical analysis of the fluorescence values of the tumors monitored on day 7 (*n* = 3/group). **D**, **E** H&E staining and statistical analysis of G422^TN^-tumor in control and PLX3397 groups on day 5, 7, and 9 (*n* = 4/group). Scale bar, 1000 μm. **F** The Kaplan-Meier survivals of G422^TN^-mice with PLX3397 therapy starting on day 1 (*n* = 8/group). **G** The volcano plot of PLX3397 treatment vs. control showing differentially expressed genes in G422^TN^-tumors (*n* = 3/group). **H** The top 20 differential signaling pathways between the Control and PLX3397 identified by the KEGG enrichment analysis. **I**–**P** Ki-67 staining (*n* = 9/group) Scale bar, 1000 μm (left), 100 μm (right) **(I)** H&E staining (calculating invasive index, *n* = 4/group) Scale bar, 500 μm (left), 50 μm (right) (**J**) Vimentin staining (*n* = 4/group) Scale bar, 1000 μm (left), 50 μm (right) (**K**) TUNEL staining, Scale bar, 1000 μm (left), 200 μm (right). **L** cleaved caspase3 staining (*n* = 4/group) (**M**) Scale bar, 100 μm (left), 50 μm (right). p-PI3K staining (*n* = 4/group) Scale bar, 100 μm (left), 50 μm (right) (**N**) p-AKT staining (*n* = 5/group) Scale bar, 100 μm (left), 50 μm (right) (**O**) and their statistical analysis (**P**) of G422^TN^-tumors on day 7 in control and PLX3397 group. *P < 0.05, **P < 0.01, ***P < 0.001, **** *P* < 0.0001.

Anti-CSF-1R therapy by using CSF-1R inhibitors alone, such as PLX3397 (Plx, i.e., Pexidartinib) prominently extends animal survival, including long-term survival in previous preclinical glioma/GBM mouse models [18, 19], which are far away from the results of anti-CSF-1R monotherapy in GBM clinical trials [44]. In the G422^TN^-GBM model, TMZ monotherapy shows the most effectiveness but can only extend animal survival for 7–8 days [22], which reflects the rapid GBM relapse. Indeed, PLX3397 monotherapy starting at day 1 post-G422^TN^-cell inoculation (p.i.) prominently reduced G422^TN^-tumor growth on day 7 p.i. (Fig. 2A–E) and on day 9 of tumor progression (Supplementary Fig. 2A, B). PLX3397 therapy killed around 90% TAMs (Supplementary Fig. 2C, D), reduced the expression of phagocytic markers on day 7 p.i. (Supplementary Fig. 2E–J) and effectively suppressed G422^TN^-tumor growth from day 5 to 7 p.i. as compared to the vehicle control (Ctrl) group (Fig. 2D, E). However, from day 7 to 9 p.i., G422^TN^-tumor growth evidently accelerated although PLX3397 therapy continued (Fig. 2D, E), suggesting the start of rapid tumor recurrence. Finally, PLX3397 monotherapy could only prolong the median survival of G422^TN^-mice for 2 days (Fig. 2F).

To explore the therapeutic mechanisms of PLX3397, we performed bulk RNA-seq with intracranial G422^TN^-tumor tissue on day 7 (Supplementary Fig. 3A). PLX3397 therapy prominently altered gene expression compared to its Ctrl (Fig. 2G). KEGG enrichment analysis showed that top 2 altered signaling pathways were Cell cycle and Phagosome (Fig. 2H). By comparing gene expression between the two groups of tumor samples (Plx vs. Control), we identified differentially expressed genes (DEGs) (Supplementary Fig. 3B). GO enrichment analysis showed that many altered top 20 pathways were associated with immune response (Supplementary Fig. 3C). GSEA revealed that PLX3397 downregulated many cell proliferation signaling pathways and the microglia phagocytosis pathway (Supplementary Fig. 3D–H). Reducing cell cycle signaling may contribute largely to PLX3397’s therapeutic effect, and reduced phagocytosis and immune response largely reflect the TAM reduction. Finally, we verified the therapeutic mechanisms of PLX3397 via immunohistochemistry, and the results demonstrated that PLX3397 prominently reduced cell proliferation (Ki67 staining), increased apoptotic cell death/signaling (TUNEL staining, cleaved caspase-3), reduced GBM cell invasion (H&E staining, Vimentin) and CSF1R-downstream signaling pathway p-PI3K/p-AKT [45, 46] (Fig. 2I–P).

### PLX3397-combined TMZ therapy synergistically extends G422^TN^-mice survival

**Fig. 3:**
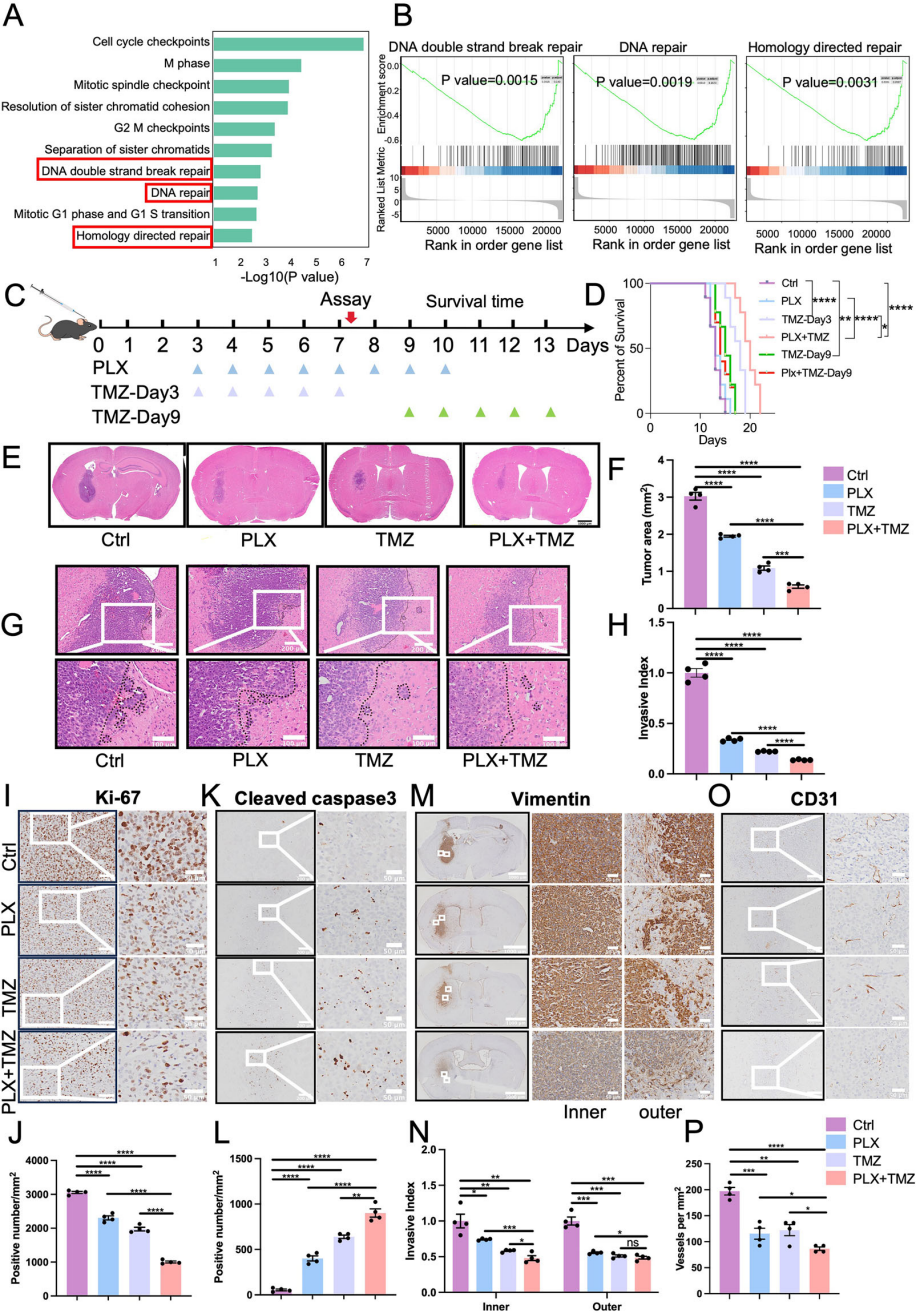
Synergistic therapeutic effects of PLX3397-combined temozolomide on G422^TN^ tumor. **A** The top 10 differential pathways between the Control and PLX3397 identified by GSEA enrichment analysis. **B** Enrichment plot of ranked genes illustrating the enrichment of DNA double strand break repair, DNA repair and homology directed repair in PLX3397 group vs. control. **C** Schematic diagram depicting the Plx, TMZ, or their combined therapy starting on day 3 or day9 (Plx 100 mg/kg/d, TMZ 30 mg/kg/d; p.o., 5 doses). **D** The Kaplan-Meier survivals of the G422^TN^-mice with different therapy starting on day 3 or day 9 (*n* = 9/group). **E**, **F** H&E staining (calculating tumor area) and statistical analysis of G422^TN^-tumor of day 7 (*n* = 4/group. Scale bar, 1000 μm. **G**–**P** =H&E staining and their statistical analysis (*n* = 4/group), Scale bar, 200 μm (up), 100 μm (down) (**G**, **H**) Ki-67 staining and their statistical analysis (*n* = 4/group), Scale bar, 100 μm (left), 50 μm (right) (**I**, **J**) cleaved caspase3 staining and their statistical analysis (*n* = 4/group), Scale bar, 200 μm (left), 50 μm (right) (**K**, **L**) vimentin staining and their statistical analysis (*n* = 4/group), Scale bar, 1000 μm (left), 50 μm (middle), 50 μm (right) (**M**, **N**) CD31 staining staining and their statistical analysis (*n* = 4/group), Scale bar, 200 μm (left), 50 μm (right) (**O**, **P**) of G422^TN^-tumor of day 7. *P < 0.05, **P < 0.01, ***P < 0.001, **** *P* < 0.0001.

Since G422^TN^-mice benefited little in survival from PLX3397 monotherapy, even starting on day 1 p.i., it is imperative to seek a synergistic drug to improve survival. Thorough GSEA analyses on bulk RNA-seq data of PLX3397-treated G422^TN^-GBM identified many DNA damage repair pathways to be among the top 10 or top 30 altered pathways (Fig. 3A, B and Supplementary Fig. 4A, B), which were downregulated in PLX3397 group (vs Ctrl group) (Fig. 3B). It is well known that TMZ therapy causes drug-resistance mainly via inducing DNA damage repair machinery [47]. Thus, we proposed that PLX3397-downregulated DNA damage repair signaling can sensitize TMZ, the only first-line drug for GBM.

We were then eager to verify the synergistic efficacy of PLX3397-combined TMZ regimen in orthotopic G422^TN^-GBM mouse model. To better evaluate synergistic efficacy, we increased therapeutic difficulty by postponing starting therapeutic time point to day 3 p.i. (Fig. 3C). Consistent with previous results, TMZ monotherapy (30 mg/kg/d) was the most effective monotherapy and extended G422^TN^-mice survival for 7 days; while PLX3397 monotherapy starting on day 3 could not extend G422^TN^-mice survival (Fig. 3D). As expected, compared to TMZ or PLX3397 monotherapy, PLX3397 + TMZ significantly prolonged G422^TN^-mice survival when TMZ therapy starting on day 3; however, PLX3397 + TMZ could not extend G422^TN^-mice survival when TMZ therapy starting on day 9 (Fig. 3D). Also, results of H&E staining and statistical analysis demonstrated that PLX3397 or TMZ monotherapy prominently reduced G422^TN^-tumor size and invasion compared to Ctrl, and that PLX3397 + TMZ further significantly reduced G422^TN^-tumor size and invasion compared to their monotherapy (Fig. 3E–H). Further immunohistochemistry study demonstrated that PLX3397 + TMZ combined therapy significantly decreased cell proliferation (Ki-67), invasion (Vimentin), angiogenesis (CD31), and increased apoptosis (cleaved caspase-3) in G422^TN^-tumor compared to their monotherapy or Ctrl groups (Fig. 3I–P). These results together support that PLX3397 can improve TMZ’ efficacy in GBM therapy.

### Identification of reduced oxidative phosphorylation in PLX3397-resistance initiation via TRAP-seq

Although PLX3397 + TMZ had evidently synergized effects in suppressing GBM’ progression initially, the median survival of G422^TN^-mice was prolonged for only 2 days compared to TMZ monotherapy (Fig. 3D). In clinical trials, PLX3397 + TMZ therapy was also only effective in a small subset of GBM patients [16, 48]. The poor survival improvement prompts us to further explored key drug-resistance mechanisms against PLX3397 therapy in GBM.

**Fig. 4:**
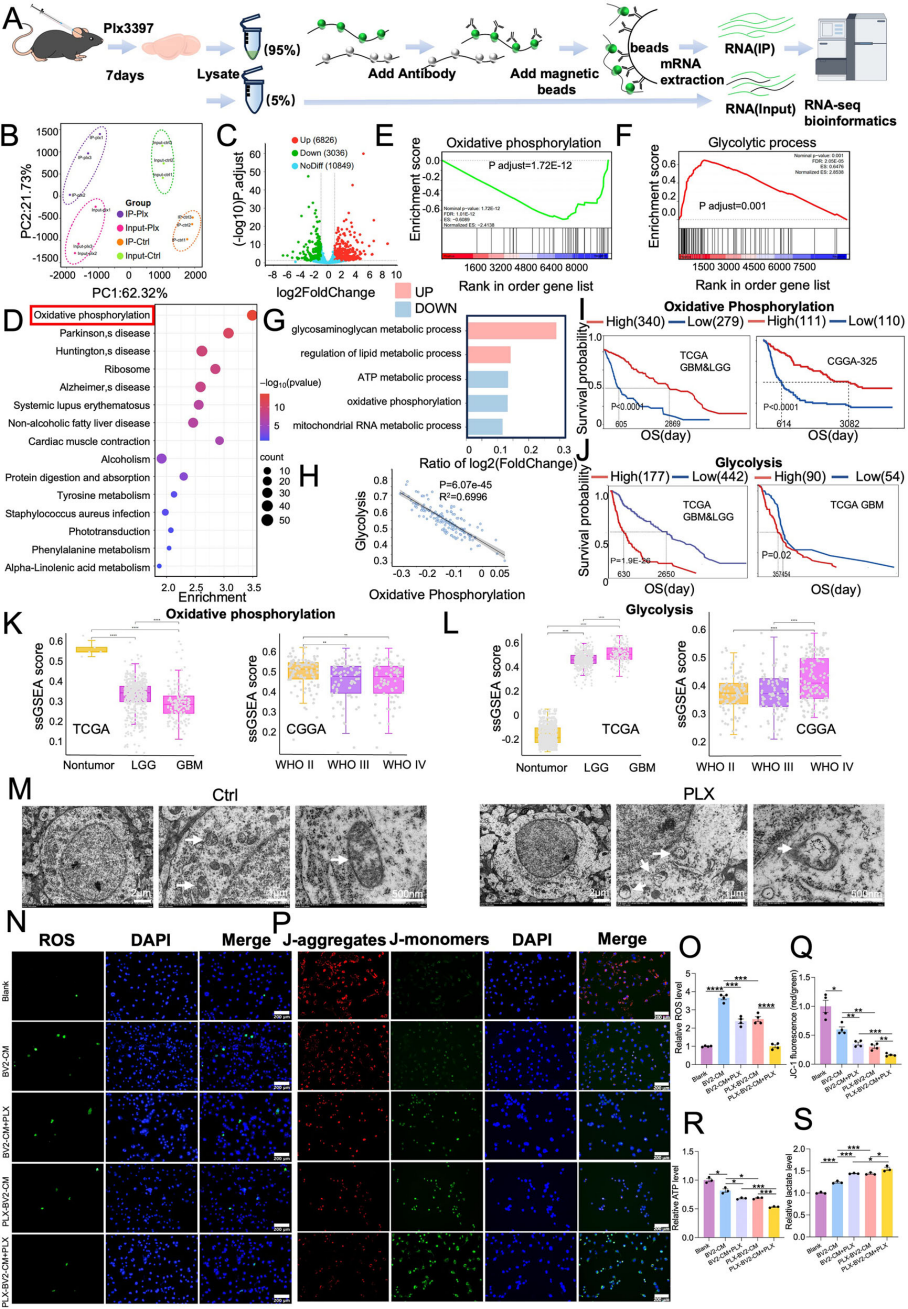
Identification of oxidative phosphorylation/glycolysis strongly associating to PLX3397-therapy resistance via TRAP-Seq. **A** Strategy for TRAP-seq of G422^TN^-GBM treated by PLX3397 and control. **B** The PCA plot of TRAP-seq data in control and PLX3397 group. **C** The volcano plot of gene expression in IP-Plx/Input-Plx vs IP-Ctrl/Input-Ctrl groups. **D** The top 15 differential pathways between the Control and Plx identified by the KEGG pathway enrichment analysis. **E** Enrichment plot of ranked genes illustrating the enrichment of oxidative phosphorylation in PLX3397 vs. control groups. **F** Enrichment plot of ranked genes illustrating the enrichment of glycolytic process in PLX3397 vs. control groups. **G** The top 5 metabolism signaling pathways with high survival probability vs. low survival probability in TCGA LGG&GBM dataset. **H** Correlation between the oxidative phosphorylation and the estimated ratios of glycolysis/gluconeogenesis in TCGA LGG&GBM dataset. **I** Patient survival with high oxidative phosphorylation level vs. low oxidative phosphorylation level in TCGA LGG&GBM or CGGA databases. **J** Patient survival with high glycolysis level vs. low glycolysis level in TCGA LGG&GBM or GBM databased. **K** The boxplot illustrating ssGSEA score of the oxidative phosphorylation in different grade glioma in TCGA dataset (Student’s t-tests) (left panel) or CGGA dataset (right panel). **L** The boxplot illustrating ssGSEA score of glycolysis in different grades of glioma in TCGA dataset (left panel) or CGGA dataset (right panel). **M** Electron micrograph of control and PLX3397 group. Scale bar, 2 μm (left), 1 μm (middle), 500 nm (right). **N**–**Q** Representative results and statistical analysis of ROS staining (**N**, **O**) and JC-1 staining (**P**, **Q**) in G422^TN^-cell co-cultures. Scale bar, 200 μm. Statistical analysis of cellular ATP (**R**) and lactate level (**S**) in G422^TN^-cell co-cultures. *P < 0.05, **P < 0.01, ***P < 0.001, **** *P* < 0.0001.

Due to the lack of valuable clue for solving PLX3397’ resistance from bulk RNA-seq in previous studies, we decided to perform TRAP sequencing on G422^TN^-cells. TRAP-seq has been widely used for studying transient gene expression in specific cell types including neuron and glia cells [49–51], but scarcely used in cancer cells. Ribosome-targeted L10a-GFP fusion protein was stably overexpressed in G422^TN^-cells (L10a-GFP/G422^TN^-cells) via lentiviral infection (Supplementary Fig. 5A) and verified by Western blotting analysis (Supplementary Fig. 5B). Subsequently, L10a-GFP/G422^TN^-cells were used to establish orthotopic GBM mouse model, and TRAP-seq was performed on day 7 post PLX3397 monotherapy (Supplementary Fig. 5C). Ribosome-attached mRNAs in L10a-GFP/G422^TN^-cells were purified by GFP antibody immunoprecipitation (IP), quality verified and then subjected to sequencing (including input lysate sample) (Fig. 4A and Supplementary Fig. 5D). Visualization of the overall gene expression revealed significant differences among IP-Plx, Input-Plx, IP-Ctrl and Input-Ctrl (Fig. 4B), indicating different changes between translating and total mRNAs after PLX3397 therapy.

Then, we compared normalized IP-Plx data (i.e., IP-Plx/Input-Plx) to that of IP-Ctrl (IP-Ctrl/Input-Ctrl). The results showed that IP-Plx had 6826 upregulated genes and 3036 downregulated genes (Fig. 4C). KEGG enrichment analysis identified oxidative phosphorylation to be the most significantly altered pathway on day 7 after PLX3397 therapy (Fig. 4D). GO enrichment analysis indicated that the top 30 altered pathways in IP-Plx were mainly associated with mitochondrial respiratory chain and mitochondrial complexes (Supplementary Fig. 5E). GSEA enrichment analysis revealed that the oxidative phosphorylation pathway was significantly downregulated in IP-Plx (Fig. 4E), while glycolysis pathway was significantly upregulated in IP-Plx and significantly inversely correlated to the change of oxidative phosphorylation (Fig. 4E, F), indicating a transition from mitochondrial respiration to glycolysis in surviving G422^TN^-cells upon PLX3397 therapy. In human GBM TCGA data, KEGG analysis revealed that oxidative phosphorylation was among the top 5 significantly altered metabolic pathways and significantly downregulated in GBM patients with longer survival time (Fig. 4G). In human GBM, ssGSEA scores of oxidative phosphorylation pathway were also significantly inversely correlated to that of Glycolysis pathway (Fig. 4H). Survival analysis with GBM patient samples in TCGA and CGGA databases showed that the higher level of oxidative phosphorylation was significantly associated with longer OS (Fig. 4I), while the higher level of glycolysis was significantly associated with shorter OS (Fig. 4J). In addition, glioma grade or malignance was negatively correlated to oxidative phosphorylation ssGSEA scores but positively correlated to glycolysis ssGSEA scores in either TCGA or CGGA database (Fig. 4K, L). Such evidence strongly suggested that G422^TN^-cells acquired PLX3397’ resistance via decreasing oxidative phosphorylation and increasing glycolysis.

The prominently altered oxidative phosphorylation/glycolysis in in PLX3397-treated G422^TN^-cells indicates impaired mitochondrial respiration. To verify this, we first examined G422^TN^-cells’ mitochondria in tissues by using transmission electron microscopy. Results clearly showed that mitochondrial damages (e.g., fragmented cristae, matrix swelling) were more common and severe in PLX3397-treated group (Fig. 4M). Then, we examined the effects of PLX3397 on G422^TN^-cell’ mitochondrial and metabolic functions in an in vitro co-culture system. G422^TN^-cells were treated with PLX3397 for 24 h and then tested the proliferation and apoptosis of tumor cells (Supplementary Fig. 6A–C). BV2 cells (mouse microglia) were treated with 10 μM of PLX3397 for 24 h, and the conditioned medium (CM) was collected (Plx-BV2-CM, and its control BV2-CM) (Supplementary Fig. 7A, B). Then, we treated G422^TN^-cells with BV2-CM/Plx-BV2-CM alone or plus PLX3397 for 24 h and performed various assays related to mitochondrial functions (Supplementary Fig. 7C). The results demonstrated that BV2-CM incubation not only prominently increased ROS, but also affected mitochondrial potential (JC-1), ATP and lactate production in G422^TN^-cells; PLX3397 significantly reduced G422^TN^-cell’ ROS/JC-1/ATP but increased lactate either directly or via BV2 cells indirectly (Fig. 4N–S). Such evidence supported that PLX3397 therapy impaired mitochondrial function, reduced oxidative phosphorylation and increased glycolysis in G422^TN^-cells in vivo.

### Discovery of drug candidates targeting oxidative phosphorylation/glycolysis

**Fig. 5:**
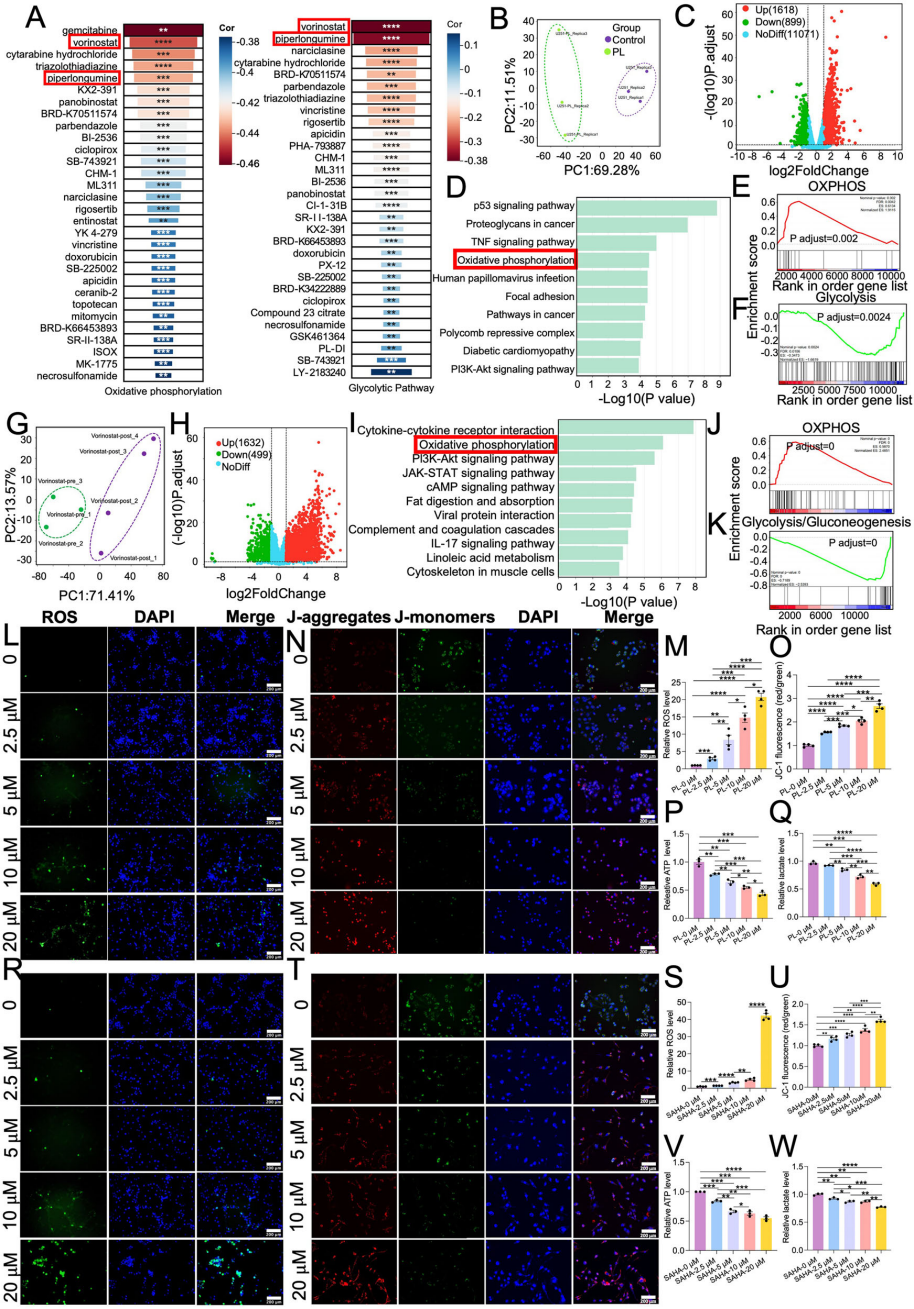
TRAP-seq combining CTRP platform identify drugs targeting to phosphorylation/glycolysis. **A** The drug lists identified from CTRP using DE genes in either oxidative phosphorylation (left) or glycolytic process (right) from TRAP-seq. **B**–**F** Bulk RNA-seq data analyses from PL-treated U251 cells. The PCA plot of PL and control data (**B**). The volcano plot of DE genes (PL vs. control) (**C**). The top 10 differential pathways between Control and PL groups identified by the KEGG pathway enrichment analysis (**D**). Enrichment plot of ranked genes illustrating the enrichment of oxidative phosphorylation (**E**) or glycolysis (**F**) (PL vs. control). **G**–**K** Bulk RNA-seq data analyses from GBM patient’ samples (Ctrl group, before therapy; Vorinostat group, after therapy). The PCA plot of Ctrl and vorinostat groups (**G**). The volcano plot of DE genes (vorinostat vs Ctrl) (**H**). The top 10 differential pathways by the KEGG pathway enrichment analysis (**I**). Enrichment plot of ranked genes illustrating the enrichment of oxidative phosphorylation (**J**) and glycolysis (**K**) (vorinostat vs. Ctrl). **L**–**Q** PL elevates ROS (**L**-**M**), JC-1 aggregates (**N**, **O**) decreased ATP level (**P**) and lactate level (**Q**) in G422^TN^-cells. (**R**–**W**) SAHA elevates ROS (**R**, **S**) JC-1 aggregates (**T**, **U**) decreased cellular ATP level (**V**) and lactate level (**W**) in G422^TN^-cells. Scale bar, 200 μm. *P < 0.05, **P < 0.01, ***P < 0.001, **** *P* < 0.0001.

Our TRAP-seq data analyses revealed that the altered oxidative phosphorylation/glycolysis pathways were highly associated to G422^TN^-cell’ resistance to PLX3397 therapy. Thus, we intended to screen drugs that may reverse PLX3397-altered oxidative phosphorylation/glycolysis signaling using CTRP platform, which links drug efficacy to gene expression in cancer cell lines. We input the top 100 DE genes in oxidative phosphorylation or glycolysis pathway (listed by *p* value, *p* < 0.05, PLX3397-IP vs Ctrl-IP) into CTRP platform to obtain related sensitive drugs. Results showed that vorinostat (SAHA), PL and cytarabine were among the top 5 listed drugs in both data sheets (Fig. 5A). Since SAHA and PL can penetrate blood blood-brain barrier while cytarabine does not, we selected SAHA and PL for further study. We analyzed previous bulk RNA-seq data of PL-treated human GBM U251 cells compared to their vehicle control (Fig. 5B, C): KEGG enrichment analysis showed that oxidative phosphorylation was listed the top 4 of all significantly changed pathways (Fig. 5D). GSEA analysis showed that oxidative phosphorylation signaling was significantly upregulated while glycolysis was significantly downregulated in the PL group (Fig. 5E, F). With similar analyses, we compared bulk RNA-seq data of human GBM from patients before and after SAHA therapy, and also found that oxidative phosphorylation was significantly upregulated while glycolysis was significantly downregulated in GBM tissues after SAHA therapy (Fig. 5G–K). These RNA-seq data verified the effects of PL and SAHA on oxidative phosphorylation/ glycolysis in human GBM cells.

Then, we verified the effects of PL and SAHA on oxidative phosphorylation/glycolysis in G422^TN^-cells in vitro. We determined the IC_50_ of PL and SAHA in G422^TN^-cells (PL: 20 μM/24 h or 5 μM/48 h; SAHA: 10 μM/24 h or 5 μM/48 h), and verified that both PL and SAHA showed a dosage-dependent cytotoxicity in G422^TN^-cells (Supplementary Fig. 8). We demonstrated that PL and SAHA at various concentrations (2.5, 5, 10, 20 μM) significantly increased ROS and mitochondrial membrane potential (JC-1 staining) but significantly decreased ATP and lactate in G422^TN^-cells (24 h of drug incubation; PL: Fig. 5L–Q; SAHA: Fig. 5R–W). Such evidence together demonstrated that PL and SAHA exert opposite effects to PLX3397 on regulating oxidative phosphorylation/glycolysis pathways.

### Piperlongumine or vorinostat sensitizes PLX3397 therapy via reversing oxidative phosphorylation/glycolysis

**Fig. 6:**
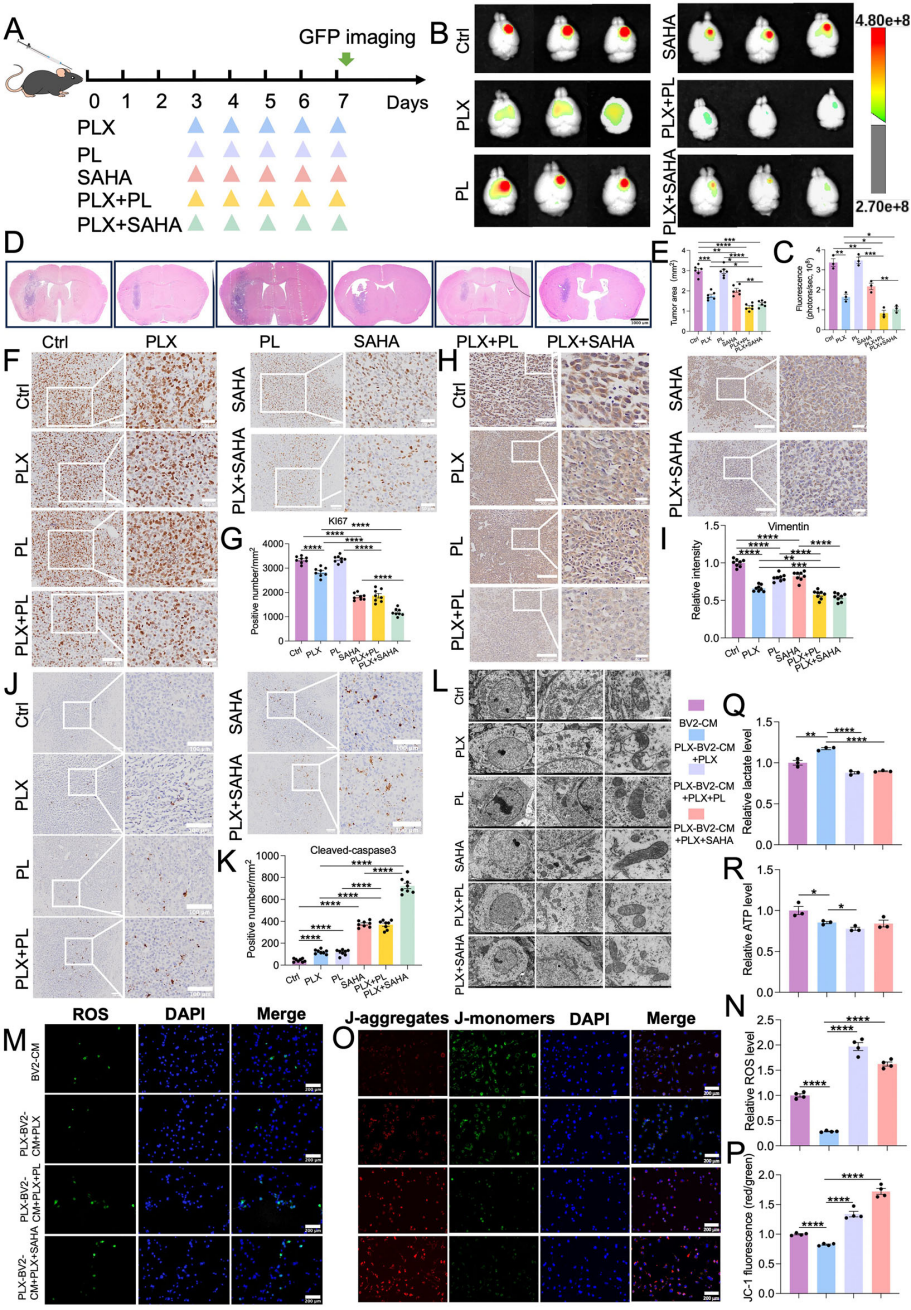
Piperlongumine or vorinostat improves PLX3397 efficacy and reverses phosphorylation/glycolysis altered by PLX3397. **A** Schematic diagram of PLX3397-combined PL or Vorinostat regimen in G422^TN^-mice. **B**, **C** Representative GFP fluorescence images and statistical analysis of G422^TN^-tumors on day 7 (*n* = 3/group). **D**, **E** H&E staining and statistical analysis of G422^TN^-tumor area (*n* = 3/group). Scale bar, 1000 μm. **F**–**K** Representative micrographs staining and statistical analysis of Ki-67 (*n* = 8/groups). Scale bar, 100 μm (left), 50 μm (right). **F**, **G** Vimentin (*n* = 4/group). Scale bar, 100 μm (left), 50 μm (right). **H**, **I** or cleaved caspase 3 (*n* = 8). Scale bar, 100 μm. **J**, **K** staining in G422^TN^-tumors on day 7. **L** Representative electron micrographs showing the mitochondrial morphology in G422^TN^-tumor after different therapy. Scale bar, 2 μm (left), 1 μm (middle), 500 nm (right). Representative micrographs and statistical analysis of ROS staining (**M**, **N**) and JC-1 staining (**O**, **P**) in G422^TN^-cell co-cultures upon various treatments. Scale bar, 200 μm. Statistical analysis of cellular ATP level (**Q**) and lactate level (**R**) upon various treatments. *P < 0.05, **P < 0.01, ***P < 0.001, **** *P* < 0.0001.

Next, we tested whether PL or Vorinostat (SAHA) can overcome G422^TN^-cell’ resistance to PLX3397 therapy and reverses PLX3397-induced oxidative phosphorylation/glycolysis alterations. Results of whole brain GFP-fluorescence imaging (GFP^+^ G422^TN^-cell) and H&E staining clearly demonstrated that either PLX3397 + PL or PLX3397 + SAHA significantly reduced G422^TN^-tumor sizes in the brain as compared to all their monotherapies after 5 days of therapy (Fig. 6A–E). Consistent with results of reduced tumor size, PLX3397 + PL or PLX3397 + SAHA significantly reduced Ki-67^+^ cells (Fig. 6F, G), vimentin expression (Fig. 6H, I), but increased cleaved-caspase3^+^ cells (Fig. 6J, K) in G422^TN^-tumor compared to their corresponding monotherapy groups. Further, electron microscopy showed less mitochondrial swelling and cristae damage in PLX3397 + PL- or SAHA + PL-treated G422^TN^-cell in tissue compared to PL monotherapy (Fig. 6L), supporting the role of PL or SAHA in reversing PLX3397-reduced oxidative phosphorylation.

After confirming the synergistic therapeutic effects of PLX3397 + PL and SAHA + PL and mechanisms in vivo, we further verified their effects on oxidative phosphorylation/glycolysis by a co-culture system in vitro. Microglia-conditioned media (BV2-CM or Plx-BV2-CM) were collected as previously described (Supplementary Fig. 7B) and were used to treat G422^TN^-cells in combination with either Plx, Plx+PL, or Plx+SAHA for 24 h (Supplementary Fig. 9). Results of ROS, JC-1, ATP, and lactate detection/measurement demonstrated that PL or SAHA prominently reversed Plx-reduced ROS level and JC-1 fluorescence, as well as Plx-elevated lactate production in Plx-BV2-CM co-incubated G422^TN^-cells, while the effect on ATP level is not significant (Fig. 6M–P). The IHC analysis revealed that PLX3397 downregulated oxidative phosphorylation markers (CYCS, MT-ND1, MT-CO1, and MT-ATP6) in G422TN tumors, whereas co-treatment with PL or SAHA partially rescued these levels (Supplementary Fig. 10). In contrast, PLX3397 upregulated glycolysis markers (GLUT1, HK2, and PKM2), with PL and SAHA reversing the elevated HK2 and PKM2 levels (Supplementary Fig. 11). These evidences together support that PL or SAHA can reverse PLX3397-altered oxidative phosphorylation/glycolysis signaling pathways and exert synergistic efficacy with PLX3397 in G422^TN^-GBM model.

### PLX3397 + TMZ + PL or PLX3397 + TMZ + SAHA regimen prominently extends survival of G422^TN^-GBM mice

**Fig. 7:**
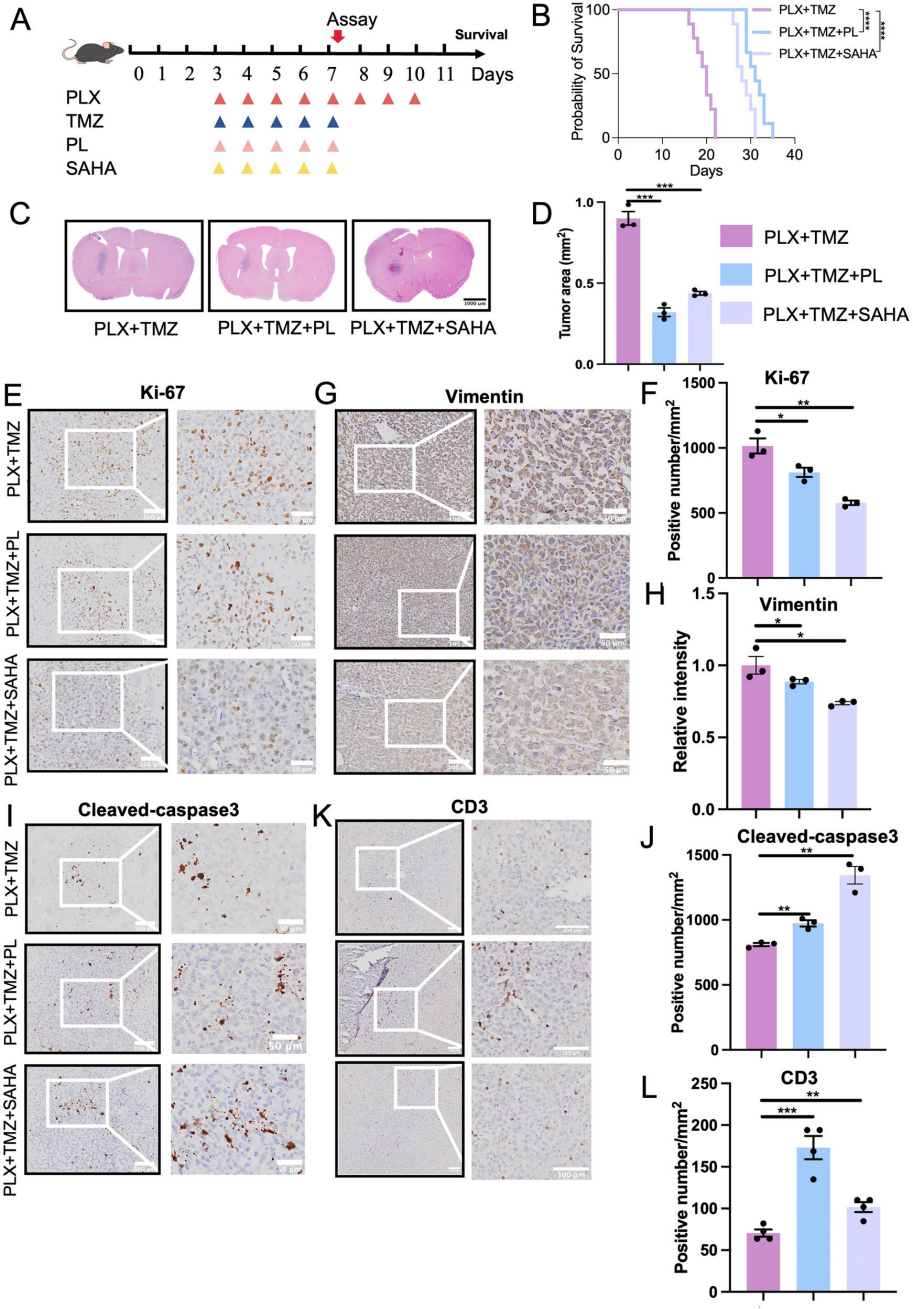
Piperlongumine or vorinostat prominently improves PLX3397 + TMZ efficacy in extending survival. **A** Schematic diagram of Plx-combined TMZ, TMZ + PL or TMZ + SAHA therapeutic regimen for H&E staining and immunohistochemistry. **B** The Kaplan-Meier survivals of the G422^TN^-mice (*n* = 7/group). **C**, **D** H&E staining and statistical analysis of G422^TN^-tumor size on day 7 (*n* = 3/group). Scale bar, 1000 μm. **E**–**L** Representative micrographs and statistical analysis of Ki-67, Scale bar, 100 μm (left), 50 μm (right). **E**, **F** Vimentin, Scale bar, 100 μm (left), 50 μm (right). **G**, **H** cleaved caspase3, Scale bar, 100 μm (left), 50 μm (right). **I**, **J** and CD3, Scale bar, 100 μm (**K**, **L**) staining (*n* = 3–4/group).

Since the synergistic efficacy of PLX3397 + PL or PLX3397 + SAHA on reduced G422^TN^-tumor size (Fig. 6D, E) did not exceed that of PLX3397 + TMZ (Fig. 3E, F), while PLX3397 + TMZ therapy could only extend median survival for 2 days in G422^TN^-GBM mice compared to TMZ monotherapy (Fig. 3H), it is conceivable that only the combination of PLX3397 + TMZ + PL or PLX3397 + TMZ + SAHA may exert longer survival prolonging effects. Thus, we tested the therapeutic efficacy of PLX3397 + TMZ + PL or PLX3397 + TMZ + SAHA regimen in G422^TN^-GBM mice (Fig. 7A–D). As expected, PLX3397 + TMZ + PL or PLX3397 + TMZ + SAHA therapy prominently reduced G422^TN^-tumor size compared to PLX3397 + TMZ group (Fig. 7C, D). Strikingly, PLX3397 + TMZ + PL or PLX3397 + TMZ + SAHA therapy for 5 days extended median survival for 10 days compared to that of PLX3397 + TMZ group, and all G422^TN^-GBM mice receiving the triple-drug regimen survived longer than those receiving PLX3397 + TMZ therapy (Fig. 7B). Consistently, PLX3397 + TMZ + PL or PLX3397 + TMZ + SAHA therapy significantly suppressed G422^TN^-cell proliferation (decreased Ki-67^+^ numbers, Fig. 7E, F) and invasion (decreased vimentin expression, Fig. 7G, H), while increased G422^TN^-cell apoptotic death (increased cleaved-caspase3^+^ cells, Fig. 7I, J) and anti-cancer immunity (increased CD3^+^ T cells, Fig. 7K, L) in G422^TN^-tumors compared to PLX3397 + TMZ group. IHC analysis revealed that PLX + TMZ + PL therapy upregulated oxidative phosphorylation markers (CYCS, MT-ND1, MT-CO1, MT-ATP6) compared to PLX + TMZ alone (Supplementary Fig. 12), while PLX + TMZ + SAHA therapy downregulated glycolysis markers (HK2, PKM2, GLUT1) in a reciprocal manner (Supplementary Fig. 13).

## Discussion

### PLX3397 evidently suppresses G422^TN^-GBM growth but ineffective in improving survival

In the highly refractory G422^TN^-GBM mouse model, we tested the therapeutic effects of anti-CSF-1R therapy by using PLX3397, which effectively killed TAMs. Starting therapy on day 1 post G422^TN^-cell inoculation (p.i.), PLX3397 evidently suppressed G422^TN^-tumor growth, leading to improved survival in the mice (Fig. 2), which is consistent with previous conclusions of preclinical study [52]. However, PLX3397 monotherapy only extended the survival of G422^TN^-GBM mice by 1–2 days, as G422^TN^-tumor relapse rapidly after 7 days of PLX3397 therapy (Fig. 2E, F). In subsequent experiments, we started PLX3397 therapy on day 3 p.i., and found that PLX3397 monotherapy had no effect on animal survival, although it remained effective in suppressing G422^TN^-tumor growth (Fig. 3), indicating the development of drug resistance. This result is similar to that observed in clinical trials, in which PLX3397 monotherapy showed poor efficacy and did not improve survival in GBM patients [44]. These results suggest that the G422^TN^-GBM model well mimics the therapeutic response of clinical patients to PLX3397, making it more suitable for exploring the mechanisms of drug resistance.

### Bulk RNA-seq identifies PLX3397 as a TMZ sensitizer in G422^TN^-GBM

Since G422^TN^-GBM develops drug-resistance to PLX3397 monotherapy rapidly, we started to seek for its effective synergistic drugs via analyzing bulk RNA-seq data from PLX3397-treated G422^TN^-tumors (Fig. 3). We found that PLX3397 mainly affected Cell cycle and Phagosome, which reflect the effects of TAMs on promoting GBM growth and PLX3397’ efficacy on killing TAMs, respectively. PLX3397-treated G422^TN^-tumors showed similar M2-like TAM’ polarization gene responses. In addition, PLX3397 downregulated most of BMDMs promoting genes and M2 polarization genes (Supplementary Fig. 14) that are associated to BLZ945’ therapeutic effects in PDG glioma model [52]. Interestingly, PLX3397 also significantly downregulated multiple DNA damage repair pathways. It is well-known that GBM cells acquire their resistance to TMZ via upregulating DNA damage repair pathways [53, 54]. Our RNA-seq data provided a rationale for PLX3397-combined TMZ therapy. TMZ is the only effective first-line drug for GBM in clinic, and is also the most effective monotherapy for improving survival in G422^TN^-GBM model (around 7 days) [22]. We speculated that PLX3397 + TMZ might exert synergistic effects and improve survival. Indeed, PLX3397 + TMZ prominently suppressed G422^TN^-tumor progression and further prolonged animal median survival (from 7 days to 9 days, vs TMZ monotherapy) (Fig. 3). This minor survival extension in PLX3397 + TMZ-treated G422^TN^-mice may explain the unsatisfactory clinical trial results, in which PLX3397 + TMZ regimen did not significantly improve GBM patient’ survival while partial therapeutic effects were observed in some patients [48, 55]. Clearly, it is imperative to find other targets/drugs that can greatly improve survival upon PLX3397 therapy.

### TRAP-seq combined CTRP analyses identifies effective target/drug to reverse PLX3397’ resistance

From bulk RNA-seq data of PLX3397-treated G422^TN^-tumors, no other evident usable target/drug that may synergize PLX3397 or reverse PLX3397’s resistance is found; thus, we switch to the TRAP-seq data for help. TRAP-seq, a ribosomal RNA-profiling or translating profiling technique, is largely applied in neuroscience but rarely applied in cancers [50]. In untreated G422^TN^-mice, G422^TN^-tumor grows rapidly with prominent tumor size increase from day 5, day 7 to day 9 p.i.; while in PLX3397-treated G422^TN^-mice, G422^TN^-tumor growth was not evident from day 5 to day 7, but rapidly enlarged from day 7 to day 9 p.i., suggesting that drug resistance occurred around day 7 in PLX3397-treated G422^TN^-mice (Fig. 2). We speculated that translating profiling by TRAP-seq on day 7 p.i. may more accurately reflect this change of surviving G422^TN^-cells and reveal the resistance mechanism of G422^TN^-cells to PLX3397 therapy.

Our TRAP-seq data analyses revealed that the most prominently altered signaling pathway was oxidative phosphorylation, which was significantly downregulated in PLX3397-treated G422^TN^-tumors (Fig. 4). In addition, PLX3397 mainly altered metabolic pathways, significantly downregulating ATP metabolic process but upregulating glycolysis signaling pathway. Such alterations suggested that surviving G422^TN^-cells were under metabolic pathways reprogramming, i.e., switching from mitochondrial respiration to glycolysis, which is highly associated with drug-resistance [56, 57]. These PLX3397-induced oxidative phosphorylation/glycolysis alterations were verified in co-culture system of G422^TN^-cells. Further, our TRAP-seq data revealed that PLX3397 upregulated PI3K-Akt signaling pathways (Supplementary Fig. 15), Stat6, IGF1r, Il4ra and Nfatc1/3/4 gene expression (Supplementary Fig. 16), which are the major resistance mechanism in previous BLZ945-treated PDG model [52, 58].

The role of reduce oxidative phosphorylation or increase glycolysis in cancer cells’ drug-resistance has been well documented [59]. Using PLX3397-altered DE genes in oxidative phosphorylation/glycolysis pathways at targeted genes (TRAP-seq data), we searched those sensitive drugs in the CTPR database that may potentially reverse PLX3397-altered oxidative phosphorylation and glycolysis (Fig. 5). PL and Vorinostat (SAHA) were the only two drugs listed among the top 5 in both oxidative phosphorylation and glycolysis pathways that can pass the blood-brain barrier. Further, PL and SAHA have been reported to exert therapeutic effects in various cancers by elevating ROS or suppressing glycolysis [60, 61]. Thus, we proposed that PL or SAHA may sensitize PLX3397 therapy by reversing oxidative phosphorylation/glycolysis. Finally, we verified the synergistic efficacy and mechanisms of PLX3397 + PL or PLX3397 + SAHA in G422^TN^-GBM model in vivo and in vitro (Fig. 6). Thus, we identified an important resistant mechanism (i.e., oxidative phosphorylation/glycolysis) and antagonistic drugs (PL and SAHA) in PLX3397 therapy. Further, our study provides an effective paradigm of TRAP-seq for identifying drug resistance mechanism in cancer.

### PL or SAHA prominently improves PLX3397 + TMZ’ efficacy in survival extension

Although PLX3397 + PL or PLX3397 + SAHA regimen exerted synergistically therapeutic effects in suppressing G422^TN^-tumor progression (Fig. 6), their efficacy did not reach that of PLX3397 + TMZ regimen (Fig. 3). This difference may be mainly caused by TMZ as TMZ has remained the most effective drug for GBM till now. In our G422^TN^-GBM model, TMZ is the most effective monotherapy in extending median survival, but the extension did not exceed 7 days; until now, we have not found any single drug in combination with TMZ that can further extend survival for more than 3 days based on TMZ therapy [21–23]. Such evidence demonstrated that our G422^TN^-GBM model is highly stable and refractory to current therapy, which mimic the therapeutic response of GBM in clinic. Thus, we speculate that only the combination of three drugs, i.e, PLX3397 + TMZ + PL or PLX3397 + TMZ + SAHA, is likely to evidently extend survival of G422^TN^-mice. Strikingly, either PL or SAHA could further extend G422^TN^-mice’ survival for 7-10 days based on PLX3397 + TMZ therapy (Fig. 7), doubling the efficacy of PLX3397 + TMZ regimen (Fig. 3). Importantly, all G422^TN^-mice receiving triple-drug therapy survived much longer than that receiving double-drug therapy, suggesting that all G422^TN^-mice can be benefited from additional PL or SAHA therapy. Mechanistically, PLX3397 + TMZ + PL regimen was more effective in increasing CD3^+^ T cells, while PLX3397 + TMZ + SAHA was more effective in suppressing GBM cell proliferation.

In summary, we identify a therapeutic mechanism (DNA repair) of PLX3397 by bulk RNA-seq, which supports the combined PLX3397 + TMZ therapy. Further, we identify a PLX3397-resistant mechanism (oxidative phosphorylation/glycolysis) by TRAP-seq, which supports the combination therapy of PLX3397 + PL or PLX3397 + SAHA. Importantly, only the triple combination of PLX3397 + TMZ + PL or PLX3397 + TMZ + SAHA prominently extends animal survival in the highly refractory G422^TN^-GBM model, which paves the next clinical trials for PLX3397-based therapy in GBM.

## Supplementary information

Supplementary materials

Uncropped Western blot

## Data Availability

Data are available for reasonable requests. Raw data were uploaded to the Sequence Read Archive (SRA) database with PRJNA1215561.
