# Supplementary material for: Identification and targeting oxidative phosphorylation/glycolysis to overcome anti-CSF-1R therapy resistance in glioblastoma
Source: Cell Death Dis. 2025 Dec 10;17(1):84. doi: 10.1038/s41419-025-08288-3 (PMC12831006; doi:10.1038/s41419-025-08288-3)
Supplement: Supplementary file 1 — Supplementary materials [file 41419_2025_8288_MOESM1_ESM.docx]

**Supplementary information**

**Identification and targeting oxidative phosphorylation/glycolysis to overcome anti-CSF-1R therapy resistance in glioblastoma**

Cheng Miao^1,2*^, Zehua Ding^2^, Jiaxing Wu^2^, Qi An^2^, Ya Shu^3^, Haifeng Jiang^2^, Panpan Gao^2^, Ruoqiao Chen^4*^, Xiao Qian Chen^2*^

^1^ Department of Obstetrics and Gynecology, Shanghai Key Laboratory of Maternal fetal Medicine, Shanghai Institute of Maternal Fetal Medicine and Gynecologic Oncology, shanghai first Maternity and infant Hospital, school of Medicine, Tongi University, Shanghai 200092, China

^2^ Department of Pathophysiology, School of Basic Medicine, Tongji Medical College, Key Laboratory of Neurological Diseases, Ministry of Education; Hubei Provincial Key Laboratory of Neurological Diseases, Huazhong University of Science and Technology, Wuhan, 430030, China.

^3^ Department of Pharmacy, The First Affiliated Hospital of Yangtze University, Jingzhou, 434000, China.

^4^ Department of Pharmacology and Toxicology, Michigan State University, East Lansing, MI 48824, USA.

***Corresponding to**: Cheng Miao, Email: [miaocheng95@foxmail.com](mailto:miaocheng95@foxmail.com); Ruoqiao Chen, Email: [chenruo4@msu.edu](mailto:chenruo4@msu.edu); Xiao Qian Chen, Email: chenxq@mails.tjmu.edu.cn

This PDF file includes:

Figures S1 to S16

**
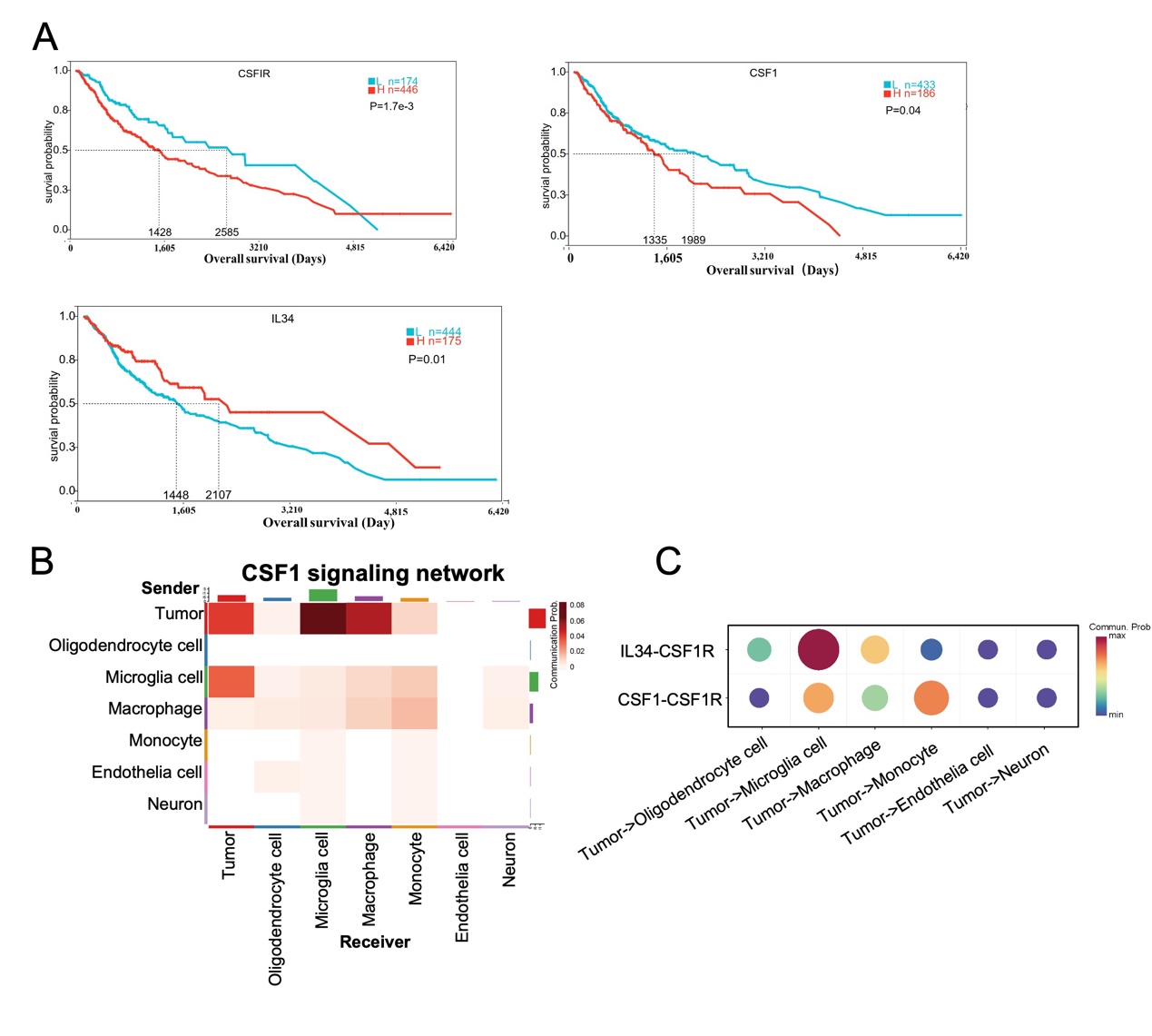
**

**Supplementary Fig. 1 Bioinformatics analysis of the CSF1 signaling pathway. (A)** Patient survival in high CSF1R expression group vs. low CSF1R expression group, high CSF1 expression group vs. low CSF1 expression group, high IL34 expression group vs. low IL34 expression group of TCGA databases. **(B)** Heatmap representation of showing the CSF signaling network in glioblastoma. **(C)** Network diagram illustrating the interactions between tumor and environment cells of CSF1 signaling pathway.

**
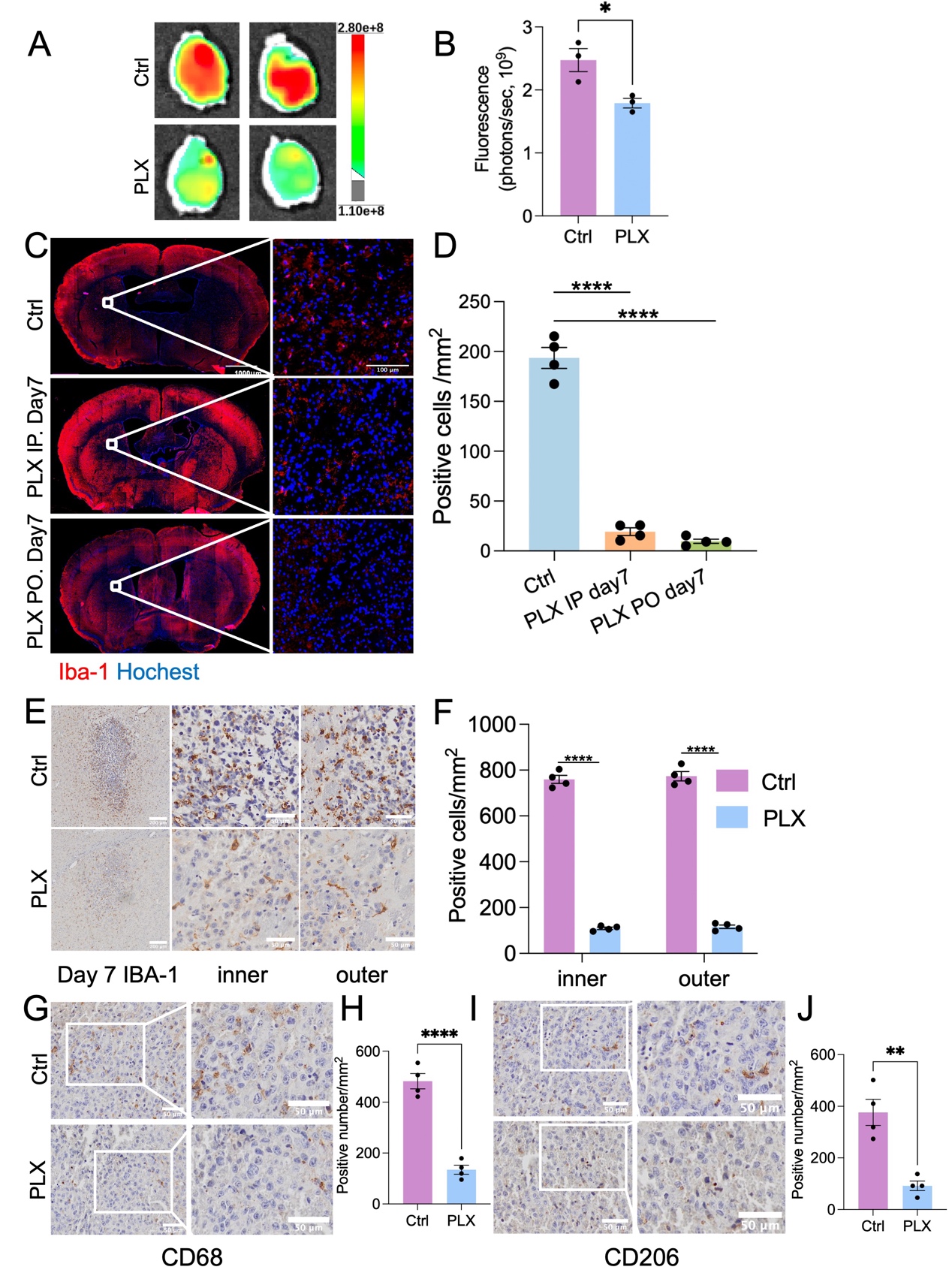
**

**Supplementary Fig. 2** **Plx3397 prominently inhibits G422^TN^-GBM growth (A-B), immunofluorescence and immunohistochemical staining determine the ablation efficiency of PLX3397 on TAMs (C-E). (A-B)** ﻿Representative GFP fluorescence images and statistical analysis of the fluorescence values of the tumors monitored on day 9 (n=3/group). **(C-D)** Immunofluorescence staining and statistical analysis of IBA-1. (n=4/group) Scale bar, 1000 μm (left), 100 μm (right). **(E-F)** Immunohistochemical staining and statistical analysis of IBA-1. (n=4/group) Scale bar, 200 μm (left), 50 μm (middle), 50 μm (right). **(G-H)** Immunohistochemical staining and statistical analysis of CD68. (n=4/group) Scale bar, 50 μm. **(I-J)** Immunohistochemical staining and statistical analysis of CD206. (n=4/group) Scale bar, 50 μm. *P<0.05, **P<0.01, ****P<0.0001.

**
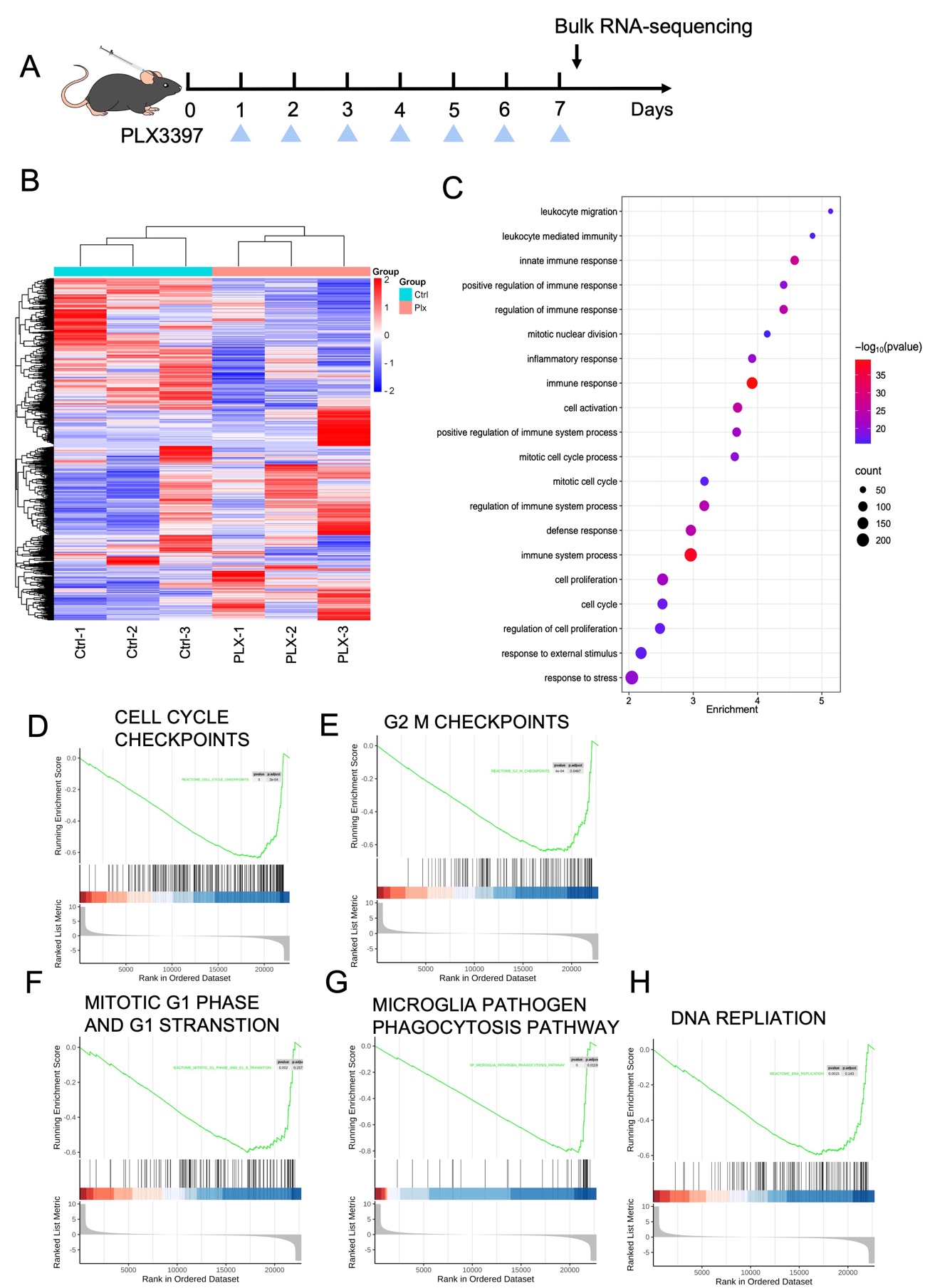
**

**Supplementary Fig. 3 Bulk RNA-seq data indicate that PLX3397 alters immune response, cell cycle and phagocytosis in G422^TN^-tumor.** **(A)** Schematic time diagram illustrating PLX3397 therapy and bulk RNA-seq. **(B)** Heatmap showing DEGs of ctrl and Plx samples. |Log2FC|>1, adjusted q value< 0.05. **(C)** ﻿The top 20 differential pathways between the Control and PLX3397 groups identified by the GO pathway enrichment analysis. **(D-H)** Enrichment plot of ranked genes illustrating the enrichment of cell cycle checkpoints **(D)**, G2 M checkpoints **(E)**, mitotic G1 phase and G1 stranstion **(F)**, and microglia pathogen phagocytosis pathway **(G)**, DNA replication **(H)** in PLX3397-treated G422^TN^-tumors vs. vehicle controls.

**
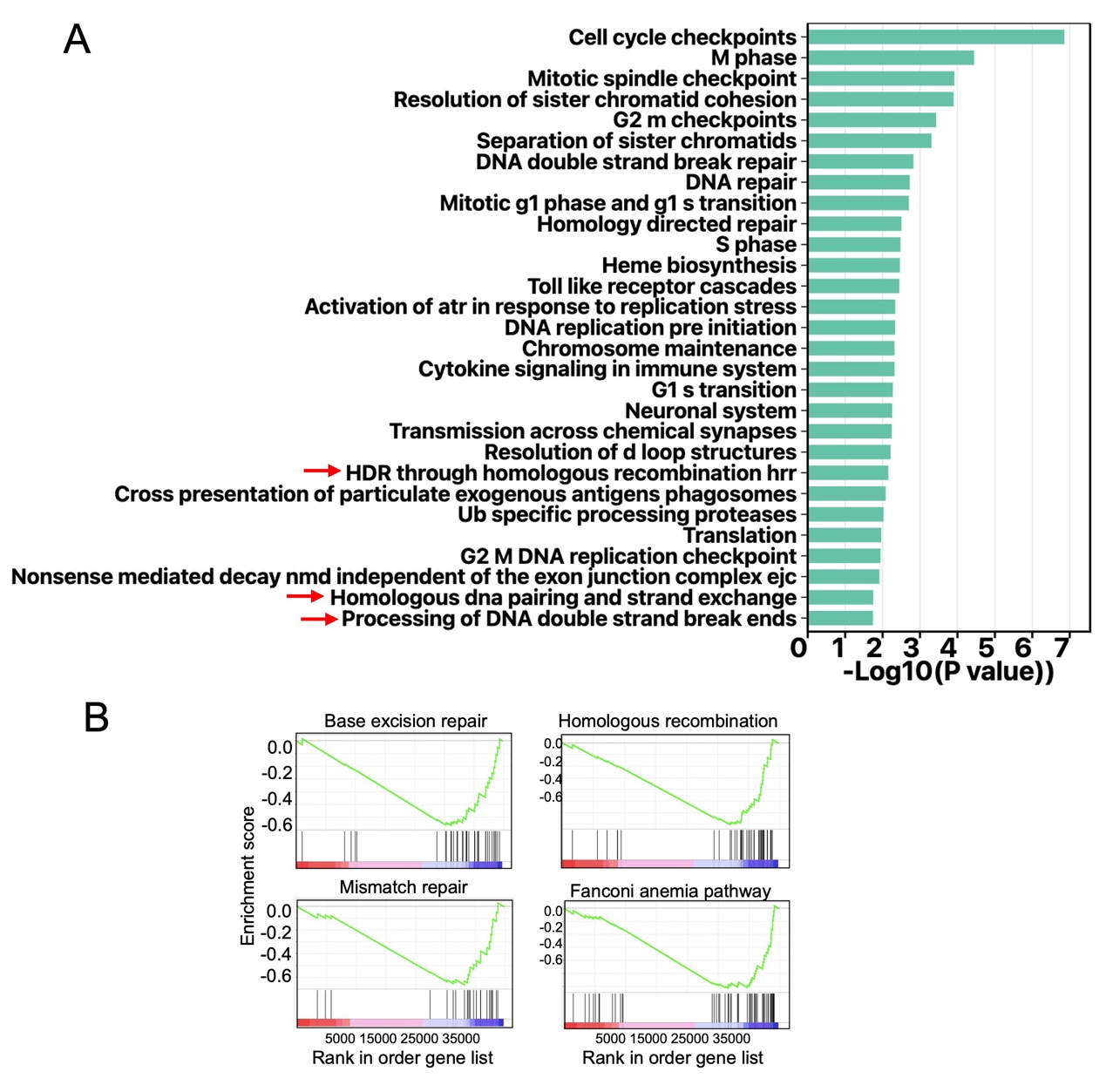
**

**Supplementary Fig. 4 Bulk RNA-seq data indicate that PLX3397 alters DNA damage repair related signals in G422^TN^-tumor. (A)** ﻿The top 30 differential pathways between the Control and PLX3397 groups identified by the GSEA pathway enrichment analysis. **(B)** Enrichment plot of ranked genes illustrating the enrichment of base excision repair, homologous recombination, mismatch repair and Fanconi anemia pathway in PLX3397-treated G422^TN^-tumors vs. vehicle controls.

**
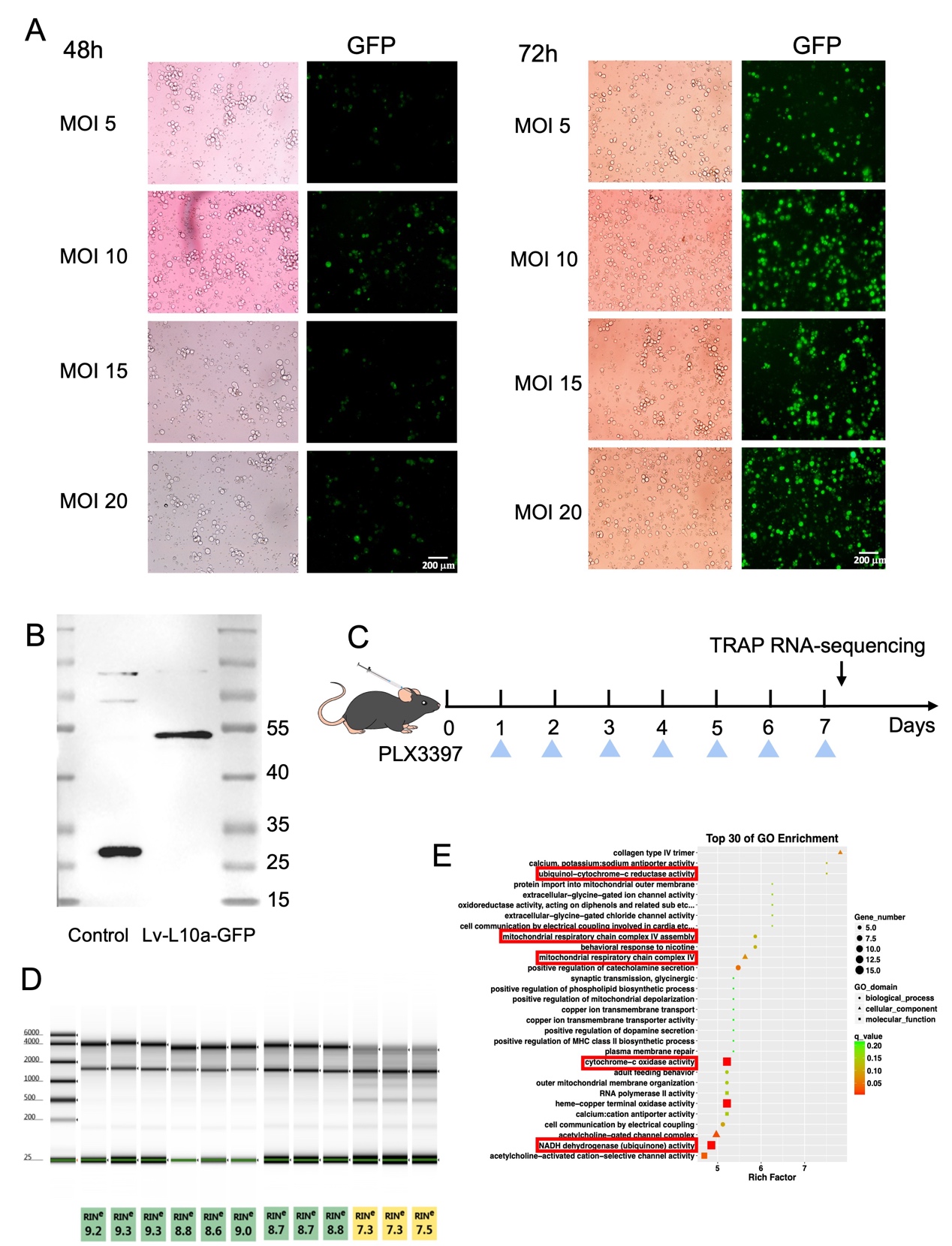
**

**Supplementary Fig. 5 Lentiviral infection and TRAP RNA-sequencing of G422^TN^-tumor cells. (A)** Determination of MOI values for lentiviral infection of G422^TN^-tumor cells. Scale bar, 200 μm. **(B)** Representative results of Western blot showing GFP (Control LV infection) and L10A-GFP (LV-L10A-GFP infection) overexpression in G422^TN^-cells. **(C)** Schematic time diagram illustrating PLX3397 therapy and TRAP RNA-seq. **(D)** Quality control chart of RNA from TRAP sequencing samples of tumor tissue. **(E)** The top 30 differential pathways between the Control and PLX3397 identified by the GO pathway enrichment analysis.

**
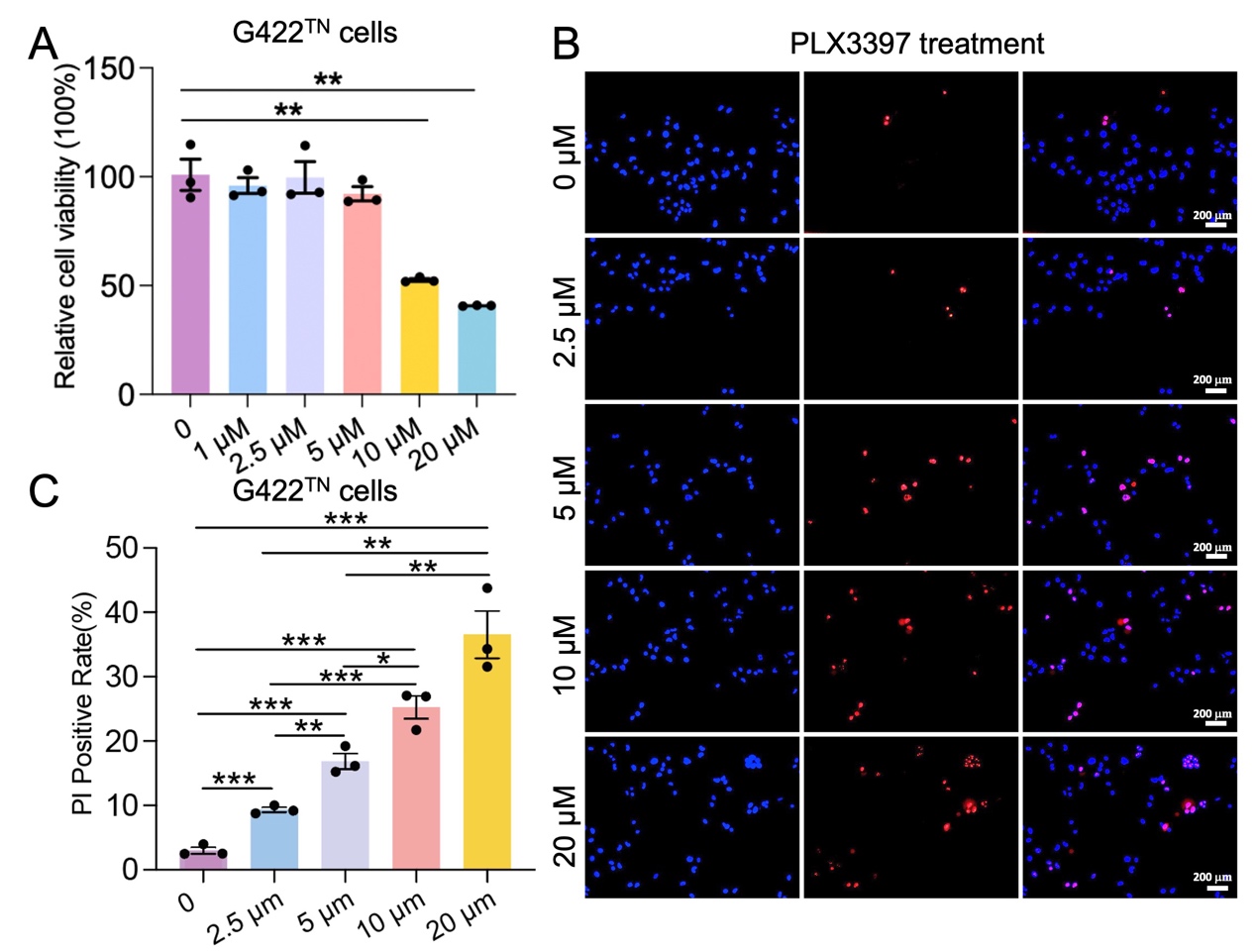
**

**Supplementary Fig. 6 PLX3397 suppresses tumor cell proliferation and induces apoptosis in vitro. (A)** CCK8 assay for the effect of PLX3397 on tumor cell viability. **(B)** PI staining and statistical analysis **(C)** for the effect of PLX3397 on tumor cell apoptosis. Scale bar, 200 μm. *P<0.05, **P <0.01, *** P<0.001, n=3/group.

**
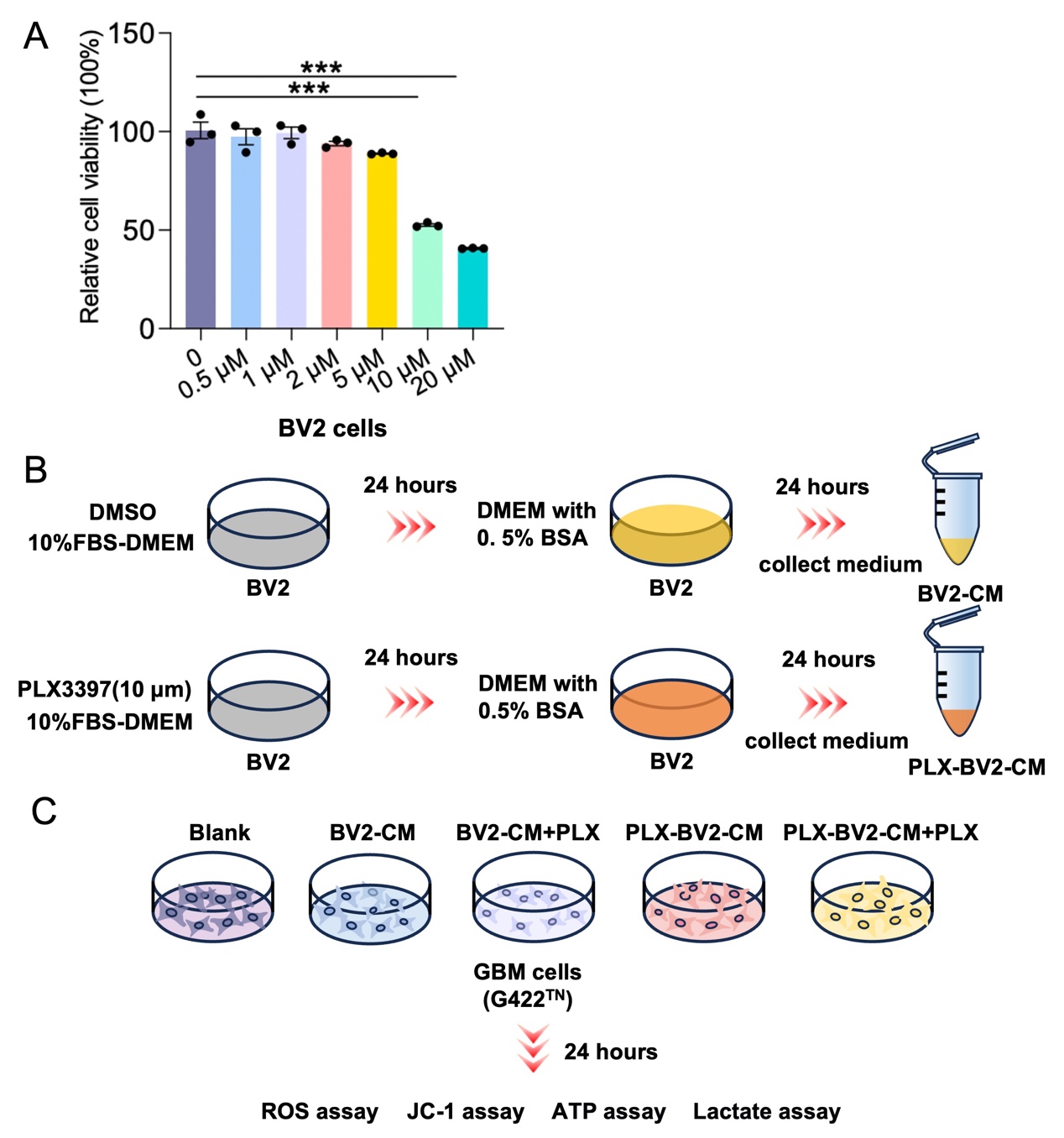
**

**Supplementary Fig. 7. Effects of PLX3397 on BV2 cell viability and the collection of conditioned media for co-culture assays. (A)** CCK8 assay for the effect of PLX3397 on BV2 cell viability. **(B)** Schematic diagram of collecting conditional medium. **(C)** Schematic representation of in vitro assays that validate the effects of PLX3397 on G422^TN^-cell’ oxidative phosphorylation/glycolysis.

**
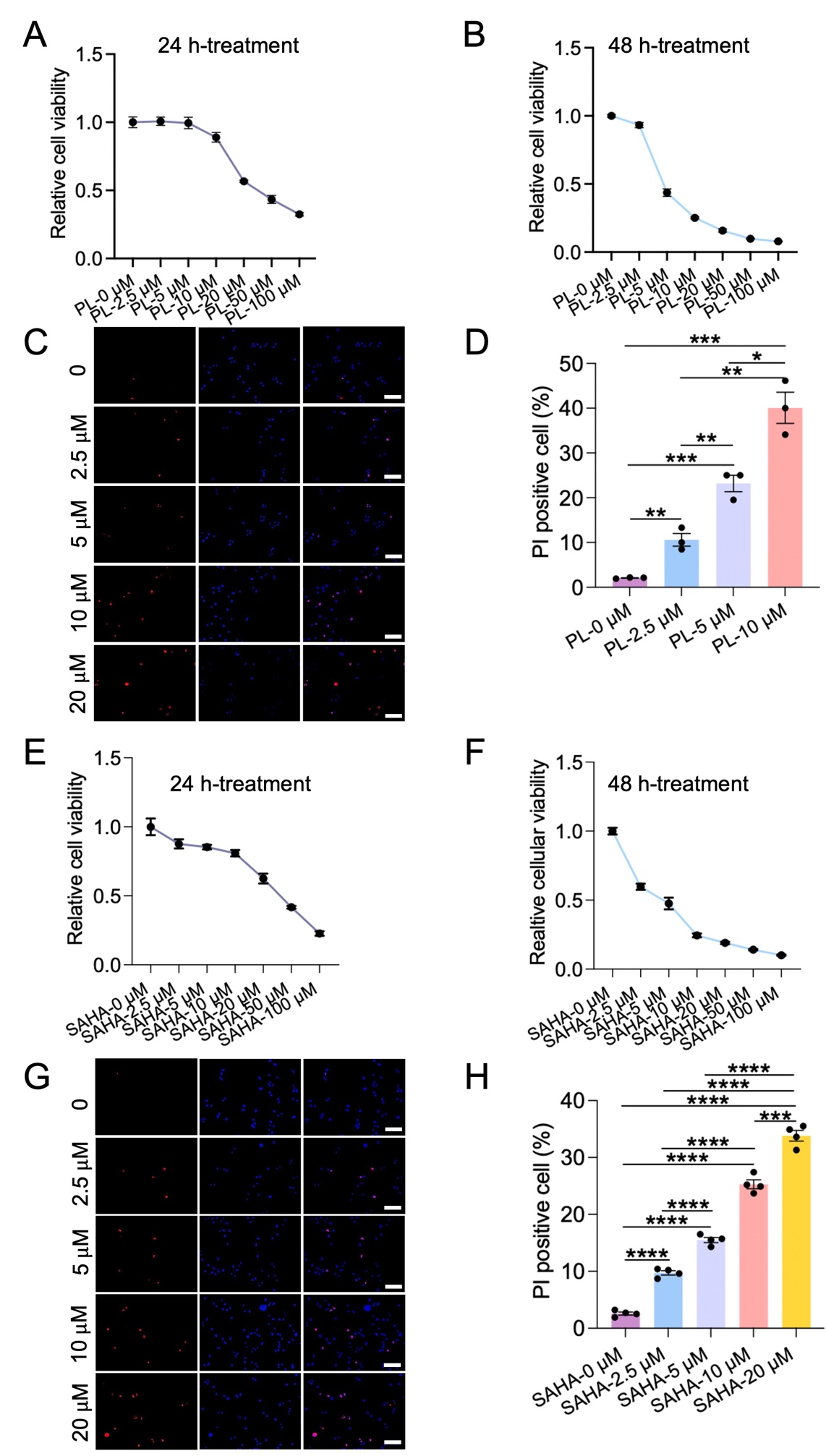
**

**Supplementary Fig. 8 PL or SAHA inhibits tumor G422^TN^-cell proliferation and promote apoptosis. (A)** CCK8 assay for the effect of PL on tumor cell viability after 24 hours treatment. **(B)** CCK8 assay for the effect of PL on tumor cell viability after 48 hours treatment. **(C)** PI staining and statistical analysis **(D)** (n=3/group) for the effect of PL on tumor cell apoptosis after 24 hours treatment. Scale bar, 200 μm. **(E)** CCK8 assay for the effect of SAHA on tumor cell viability after 24 hours treatment. **(F)** CCK8 assay for the effect of SAHA on tumor cell viability after 48 hours treatment. **(G)** PI staining and statistical analysis **(H)** for the effect of SAHA on tumor cell apoptosis after 24 hours treatment. Scale bar, 200 μm. *P<0.05, **P <0.01, ***P<0.001, **** P<0.0001, n=3/group.

**
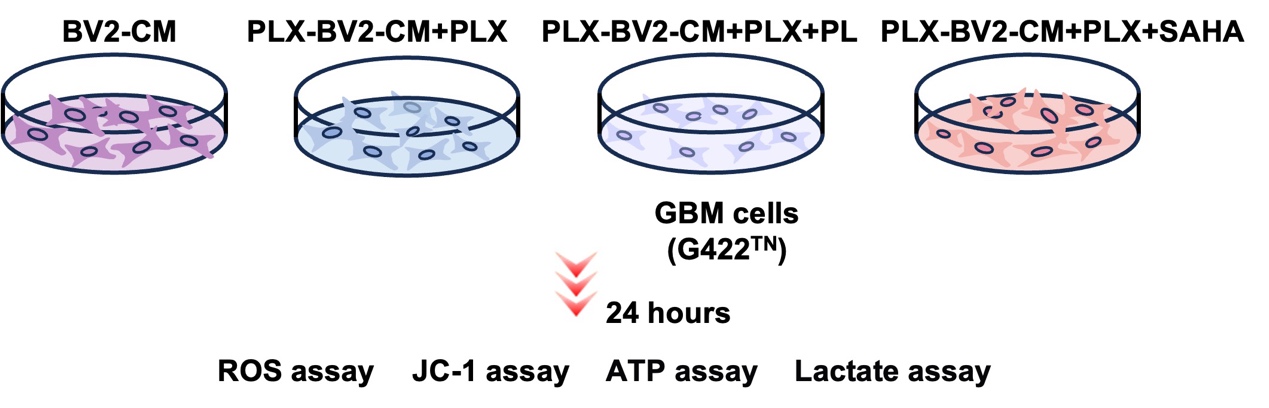
**

**Supplementary Fig. 9 Schematic representation of in vitro assays to validate the effects of PLX3397+PL/SAHA on G422^TN^-cell energy metabolism.**

**Supplementary Fig 10** **The effects of PLX3397, PL, SAHA monotherapy and combination therapy on oxidative phosphorylation levels in G422TN-tumor. (A)** Immunohistochemical staining and statistical analysis of CYCS. (n=4/group) **(B)** Immunohistochemical staining and statistical analysis of MT-ND1. (n=4/group) **(C)** Immunohistochemical staining and statistical analysis of MT-CO1. (n=4/group) **(D)** Immunohistochemical staining and statistical analysis of MT-ATP6. (n=4/group) **(E)** Immunohistochemical staining and statistical analysis of PKM2. (n=4/group) Scale bar, 50 μm. *P<0.05, **P<0.01, ***P<0.001

**Supplementary Fig 11 The effects of PLX3397, PL, SAHA monotherapy and combination therapy on glycolysis levels in G422^TN^-tumor.** **(A)** Immunohistochemical staining and statistical analysis of MCT4. (n=4/group) **(B)** Immunohistochemical staining and statistical analysis of GLUT1. (n=4/group) **(C)** Immunohistochemical staining and statistical analysis of LDHA. (n=4/group) **(D)** Immunohistochemical staining and statistical analysis of HK2. (n=4/group) **(E)** Immunohistochemical staining and statistical analysis of PKM2. (n=4/group) Scale bar, 50 μm. *P<0.05, **P<0.01, ***P<0.001, ****P<0.0001.

**Supplementary Fig 12 The effects of PLX3397+TMZ and the combination of PL and SAHA on oxidative phosphorylation levels in G422^TN^-tumor. (A-B) (A)** Immunohistochemical staining and statistical analysis of CYCS. (n=4/group) **(B)** Immunohistochemical staining and statistical analysis of MT-ND1. (n=4/group) **(C)** Immunohistochemical staining and statistical analysis of MT-CO1. (n=4/group) **(D)** Immunohistochemical staining and statistical analysis of SDHA. (n=4/group) **(E)** Immunohistochemical staining and statistical analysis of MT-ATP6. (n=4/group) Scale bar, 50 μm. *P<0.05, **P<0.01, ***P<0.001.

**Supplementary Fig 13** **The effects of PLX3397+TMZ and the combination of PL and SAHA on glycolysis levels in G422^TN^-tumor. (A)** Immunohistochemical staining and statistical analysis of HK2. (n=4/group) **(B)** Immunohistochemical staining and statistical analysis of PKM2. (n=4/group) **(C)** Immunohistochemical staining and statistical analysis of GLUT1. (n=4/group) **(D)** Immunohistochemical staining and statistical analysis of LDHA. (n=4/group) **(E)** Immunohistochemical staining and statistical analysis of MCT4. (n=4/group) Scale bar, 50 μm. **P<0.01, ****P<0.0001.

**Supplementary Fig 14** **Comparison of TAM’ regulating gene responses in G422^TN^-GBM.** RNA-seq data shows the alterations of BMDM’ promoting genes and M2 polarization genes in PLX3397-treated G422^TN^-tumors (PLX3397 vs Control).

**Supplementary Fig 15** **Comparison of resistance mechanisms in PLX3397-treated G422^TN^-tumors.** KEGG analyses of TRAP RNA-seq data show that PI3K-Akt signaling pathway was also enriched and significantly up-regulated in tumor cells after PLX3397 monotherapy (PLX3397 vs Control groups).

**Supplementary Fig 16 Analyses of Stat6, IGF1r, Il4ra and Nfatc1/3/4 expression of RAP-seq data.** Analyses of TRAP-seq data showed that Stat6, IGF1r, Il4ra and Nfatc1/3/4 were significantly upregulated in tumor cells after PLX3397 monotherapy (PLX3397 vs Control groups).
