# Supplementary figures and images for: Identification and targeting oxidative phosphorylation/glycolysis to overcome anti-CSF-1R therapy resistance in glioblastoma
Source: Cell Death Dis. 2025 Dec 10;17(1):84. doi: 10.1038/s41419-025-08288-3 (PMC12831006; doi:10.1038/s41419-025-08288-3)

Uncropped Western blot for supplementary Fig.5B


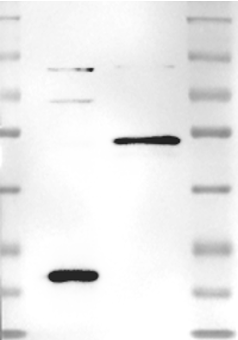


25kDa

55kDa

IB: GFP

Supplement: Supplementary file 2 — Uncropped Western blot [file 41419_2025_8288_MOESM2_ESM.docx]
